# Theoretical exploration on structures, bonding aspects and molecular docking of *α*-aminophosphonate ligated copper complexes against SARS-CoV-2 proteases

**DOI:** 10.3389/fphar.2022.982484

**Published:** 2022-10-03

**Authors:** Oval Yadav, Manjeet Kumar, Himanshi Mittal, Kiran Yadav, Veronique Seidel, Azaj Ansari

**Affiliations:** ^1^ Department of Chemistry, Central University of Haryana, Mahendergarh, India; ^2^ Natural Products Research Laboratory, Strathclyde Institute of Pharmacy and Biomedical Sciences, University of Strathclyde, Glasgow, United Kingdom

**Keywords:** DFT, molecular docking, NBO, MEP map, copper species

## Abstract

Recent years have witnessed a growing interest in the biological activity of metal complexes of *α*-aminophosphonates. Here for the first time, a detailed DFT study on five *α*-aminophosphonate ligated mononuclear/dinuclear Cu^II^ complexes is reported using the dispersion corrected density functional (B3LYP-D2) method. The electronic structures spin densities, FMO analysis, energetic description of spin states, and theoretical reactivity behaviour using molecular electrostatic potential (MEP) maps of all five species are reported. All possible spin states of the dinuclear species were computed and their ground state *S* values were determined along with the computation of their magnetic coupling constants. NBO analysis was also performed to provide details on stabilization energies. A molecular docking study was performed for the five complexes against two SARS-CoV-2 coronavirus protein targets (PDB ID: 6LU7 and 7T9K). The docking results indicated that the mononuclear species had a higher binding affinity for the targets compared to the dinuclear species. Among the species investigated, species **I** showed the highest binding affinity with the SARS-CoV-2 Omicron protease. NPA charge analysis showed that the heteroatoms of model species **III** had a more nucleophilic nature. A comparative study was performed to observe any variations and/or correlations in properties among all species.

## Introduction

Metal-based complexes containing organic ligands play crucial roles in biochemical systems including as biocatalysts, metal ions conductors across cell membranes, and new potential drugs for the treatment of various diseases (Congradyova and Jomova, 2013; Baek et al., 2018; Ali et al., 2021; Kiwaan et al., 2021). Along with the advancement of medicinal inorganic chemistry, such complexes have gained considerable interest as therapeutic agents (Ali and van Lier, 1999; Louie and Meade, 1999; Sakurai et al., 2002; Rafique et al., 2010). Complexation with metal ions has been shown to alter the pharmacological and therapeutic effects of ligands, providing new opportunities for the design of metal-based drugs in medicinal chemistry (Weder et al., 2002).

*α*-Aminophosphonates are an important class of amino acid analogues (Mucha et al., 2011) with diverse applications, in medicinal chemistry, agriculture and other industries, as synthetic intermediates with interesting pharmacological properties (Gemmill et al., 2005; Dinér and Amedjkouh, 2006; Juribasic et al., 2011; Tusek-Bozic, 2013; Boshta et al., 2018; Imam et al., 2018). They have been reported to inhibit enzymes involved in amino acid metabolism, thus having a direct influence on cell functions (Naydenova et al., 2010). Many studies have reported the remarkable activity of *α*-aminophosphonates as pharmacophores (Reddy et al., 2012; Chinthaparthi et al., 2013). These compounds exhibit potent biological properties including as plant growth regulators (Kafarski et al., 1989), antiviral (Long et al., 2008; Chen et al., 2009; Singh et al., 2020), anti-inflammatory agents (Sujatha et al., 2017; Romero-Estudillo et al., 2019), herbicides (Chen et al., 2015), protein inhibitors (Sienczyk and Oleksyszyn, 2009; Wang et al., 2012; Moreno-Cinos et al., 2019), antitumor/antiproliferative agents (Gu and Jin, 2012; Kraicheva et al., 2012; Liu et al., 2017; López-Francés et al., 2021), and antimicrobials (Atherton et al., 1986; El Sayed et al., 2011; Rezaei et al., 2011; Sudileti et al., 2019a). The biological activity of *α*-aminophosphonates has been linked with the presence of heterocyclic motifs in their structures (Sudileti et al., 2019b; Poola et al., 2019; Mohan et al., 2020). Diphenyl ester derivatives of *α*-aminophosphonates and their complexes had remarkable activity against serine proteases which play a crucial role in cancer cell disruption. Wang et al. reported that some cyclic *α*-aminophosphonates could mimic the antitumor activity of doxorubicin drug (Ma et al., 2011).

Platinum group metal-based aminophosphonate complexes have been reported to exhibit potent activity against tumour cells (Aranowska et al., 2006). A few other transition metals, like gadolinium, technetium, and rhenium have been previously used in metal-based complexes with ligands as therapeutic agents and for diagnostic purposes (Tusek-Bozic, 2013). Several *α*-aminophosphonates ligated metal complexes have been reported (Azzam et al., 2020). Derivatives of metal ligated aminophosphonates have gained huge attention due to their wide-range spectrum of bioactivities and tremendous application in the medical field (Azzam et al., 2020). Comprehensive studies have previously been carried out using copper to form copper (II) complexes with predominant applications as antiviral, anticancer and antimicrobial agents (Ranford et al., 1993; Marzano et al., 2006; Tardito et al., 2007; Repicka et al., 2009; Creaven et al., 2010; Saeed et al., 2010; Konarikova et al., 2013; Santini et al., 2014; Wehbe et al., 2017; Massoud et al., 2018). To the best of our knowledge, no computational research has been carried out on carbocyclic ring containing *α*-aminophosphonate ligands containing copper ions.

Over the past 2 years, the COVID-19 pandemic has caused a significant number of deaths worldwide. Immense efforts have been made to develop anti-COVID vaccines and antiviral drugs, but there is no effective therapeutic approach that can effectively cure this disease to date (Xu et al., 2006). *α*-Aminophosphonates have been reported to exhibit antiviral activity (Mohapatra et al., 2022). The growing interest in the biological activity of *α*-aminophosphonates metal complexes, and the reported feasibility of their large scale synthesis, motivated us to carry out an extensive computational study on these compounds with a particular focus on the interaction of metal ligation with the *α*-aminophosphonates. Our study also aimed to explore the potential of Cu^II^ *α*-aminophosphonates complexes to interact with key proteins of the COVID-19-causing SARS-CoV-2 coronavirus. Herein we report a DFT study on five *α*-aminophosphonate (L) ligated mononuclear/dinuclear Copper (II) complexes, namely species **I** [Cu^II^(L) (Br) (H_2_O)], species **II** [Cu^II^(L) (Br)_2_], species **III** [Cu^II^(L) (Cl)_2_], species **IV** [Cu_2_(HL) (Cl)_4_(H_2_O)] and species **V** [Cu_2_(HL) (Br)_2_(Cl)_2_(H_2_O)]. Among these species, **I** and **IV** have been experimentally synthesized and well characterized (Azzam et al., 2020). Species **II** and **III** are model species of **I** whereas species **V** is a model species of **IV**. Here we have addressed and discussed: electronic structures, spin state energetics, Mulliken spin densities, FMO analysis and electrostatic potentials, NBO analysis and molecular docking towards two SARS-CoV-2 protein targets (PDB ID: 6LU7 and 7T9K) with all five species followed by cross comparison among them.

### Computational details

Gaussian 16 was used to perform all geometry optimizations employing a dispersion corrected density functional B3LYP-D2 method (Frisch et al., 2016). The accuracy of the used functional was confirmed in previous studies (Ansari et al., 2013; Ansari et al., 2014; Ansari et al., 2015; Ansari et al., 2017; Yadav et al., 2021; Ansari and Monika, 2022). The LACVP basis set comprising LanL2DZ (Los Alamos effective core potential) for the copper metal centre (Dunning et al., 1977; Hay and Wadt, 1985a; Hay and Wadt, 1985b; Wadt and Hay, 1985) and a 6-31G (Ditchfield et al., 1971) basis set for the other atoms (P, H, C, N, O, Cl and Br) were employed. Frequency calculations were done on the optimized structure to confirm the minima on the potential-energy surface. The single point energy calculations were performed on optimized geometry using TZVP basis set with acetonitrile as a solvent using the polarizable continuum solvent (PCM) model (Schafer et al., 1992; Schafer et al., 1994; Tomasi et al., 2005). The quoted DFT energies are B3LYP-D2 solvation including free-energy corrections with TZVP basis set at room temperature.

## Results and discussion

### [Cu^II^(L) (Br) (H_2_O)] (species I), [Cu^II^(L) (Br) (Br)] (species II) and [Cu^II^(L) (Cl) (Cl)] (species III)

**FIGURE 1:**
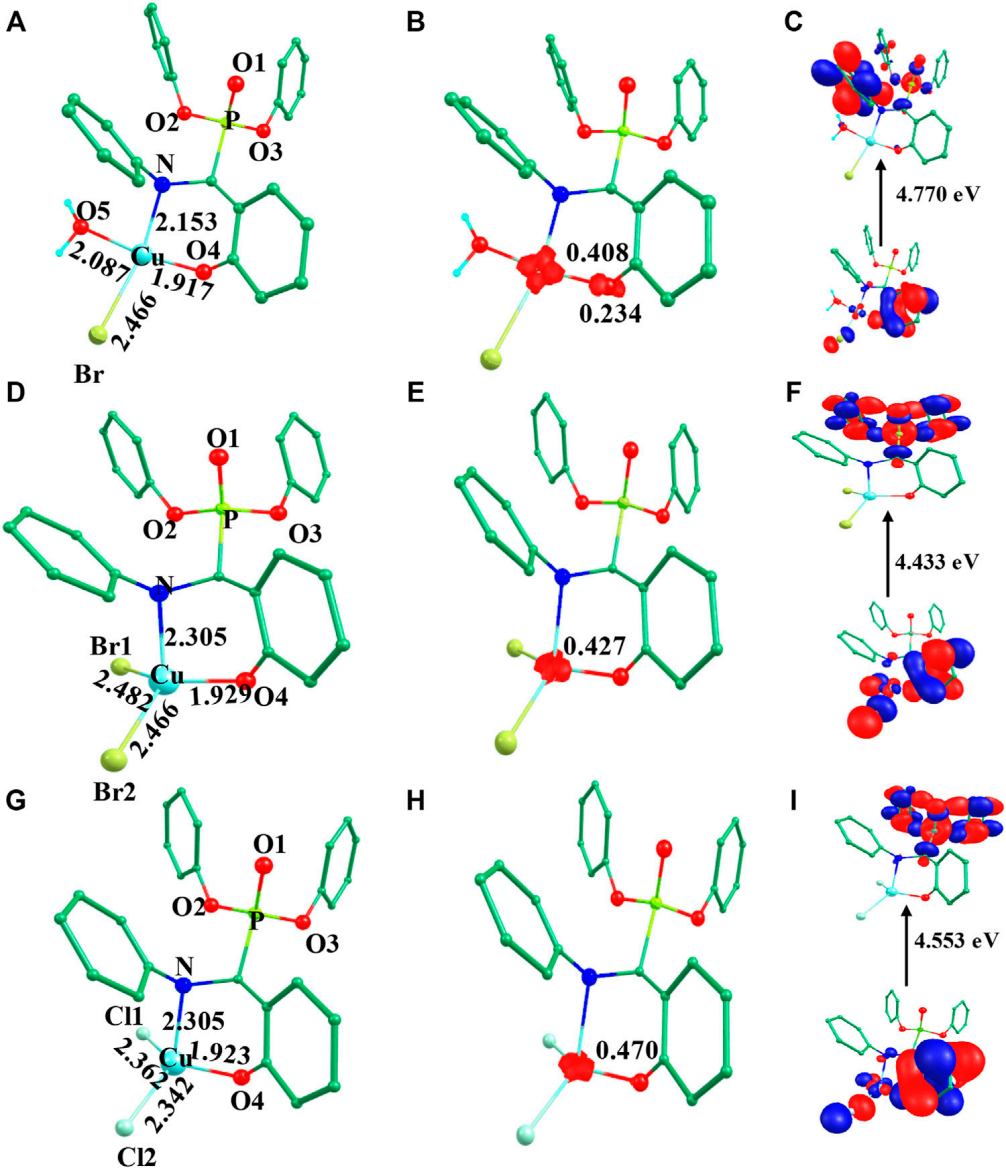
B3LYP-D2 **(A)** optimized structure of species **I** and its **(B)** spin density plot and **(C)** HOMO-LUMO gap; **(D)** optimized structure of species **II** and its **(E)** spin density plot and **(F)** HOMO-LUMO gap; **(G)** optimized structure of species **III** and its **(H)** spin density plot and **(I)** HOMO-LUMO gap. (Bond lengths are in Å).

Initially, we optimized *α*-aminophosphonate (L; see Supplementary Figure S1) and coordinated with the copper centre to generate copper [Cu^II^(L) (Br) (H_2_O)] species **I**. The latter was synthesized by the reaction of Cu^II^ salt with ligand (L) and characterized by various techniques like FT-IR and EI-Mass (Azzam et al., 2020). This species was reported recently to show activity against HT-29 cancer cell lines. The higher bioactivity of species **I** compared to the corresponding parent ligand suggested that it could be further exploited for other pharmacological applications (Azzam et al., 2020). The optimized geometry and spin density plot of species **I** are shown in Figure 1. The Cu-Br, Cu-N and Cu-O4 bond lengths were computed to be 2.466 Å, 2.153 Å and 1.917 Å, respectively. The phenyl rings of the phosphonate moiety were *cis* to each other and in the same plane. Our computed results showed that species **I** found as a distorted square planar, leaning towards tetrahedral, geometry in good agreement with experimental observations (Azzam et al., 2020). A spin density of ρ = 0.408 was located at the copper centre (see Figure 1B and Supplementary Table S1) which corresponds to unpaired electron nature at the metal centre with strong electron delocalization present in the system. The eigenvalue plot is shown in Supplementary Figure S2 and the electronic configuration at the metal centre was found to be (d_xz_)^2^ (d_yz_)^2^ (d_z_^2^)^2^ (d_xy_)^2^ (d_x_^2^_-y_^2^)^1^. The HOMO-LUMO gap for species **I** was computed to be 4.770 eV.

The previously reported remarkable bioactivity of species **I** (Azzam et al., 2020) prompted us to further study its models (species **II** and **III)**. In species **II**, the water molecule was replaced by a bromide and in species **III**, both bromides were replaced by chloride ligands. The optimized geometry and spin density plot of species **II** is shown in Figures 1D,E. Here again, a distortion of the square planar geometry, leaning towards tetrahedral, was witnessed. The distortion was expressed by bond angles of 116.4° and 100.0° for ∠Br1-Cu-Br2 and ∠Br2-Cu-O. The computed Cu-Br_avg_, Cu-N and Cu-O4 bond lengths were found to be 2.474 Å, 2.305 Å and 1.929 Å, respectively. The computed spin density of ρ = 0.427 was located at the copper centre (see Figure 1E and Supplementary Table S1) which corresponded to the unpaired electron character at the metal centre. The eigenvalue plot is shown in Supplementary Figure S3 and the electronic configuration at the metal centre was found to be (d_xz_)^2^ (d_yz_)^2^ (d_z_^2^)^2^ (d_xy_)^2^ (d_x_^2^_-y_^2^)^1^. The HOMO-LUMO gap for species **II** was computed to be 4.433 eV.

The optimized geometry and spin density plot of species **III** is shown in Figures 1G,H. The computed Cu-Cl_avg_, Cu-N and Cu-O4 bond lengths were found to be 2.352 Å, 2.305 Å and 1.923 Å, respectively. The bond angles, 87.8° and 119.5° for ∠Cl1-Cu-N and ∠Cl1-Cu-Cl2, expressed distortion in the square planar geometry of the system towards tetrahedral. The computed data suggested that both model species exhibited nearly the same distortion in their geometry and that the substitution with a halide had little effect on the geometry of the complexes. A spin density ρ = 0.470 located at the copper centre corresponding to the presence of an unpaired electron. The electronic configuration at the metal centre was found to be (d_yz_)^2^ (d_xz_)^2^ (d_z_^2^)^2^ (d_xy_)^2^ (d_x_^2^_-y_^2^)^1^ (see Supplementary Figure S4). The HOMO-LUMO gap for species **III** was computed to be 4.553 eV. This is larger than that obtained for species **II** and smaller than for species **I**.

### [(Cu^II^)_2_(L)(Cl)_4_(H_2_O)] (species IV) and [(Cu^II^)_2_(L)(Cl)_2_(Br)_2_(H_2_O)] (species V)

**FIGURE 2:**
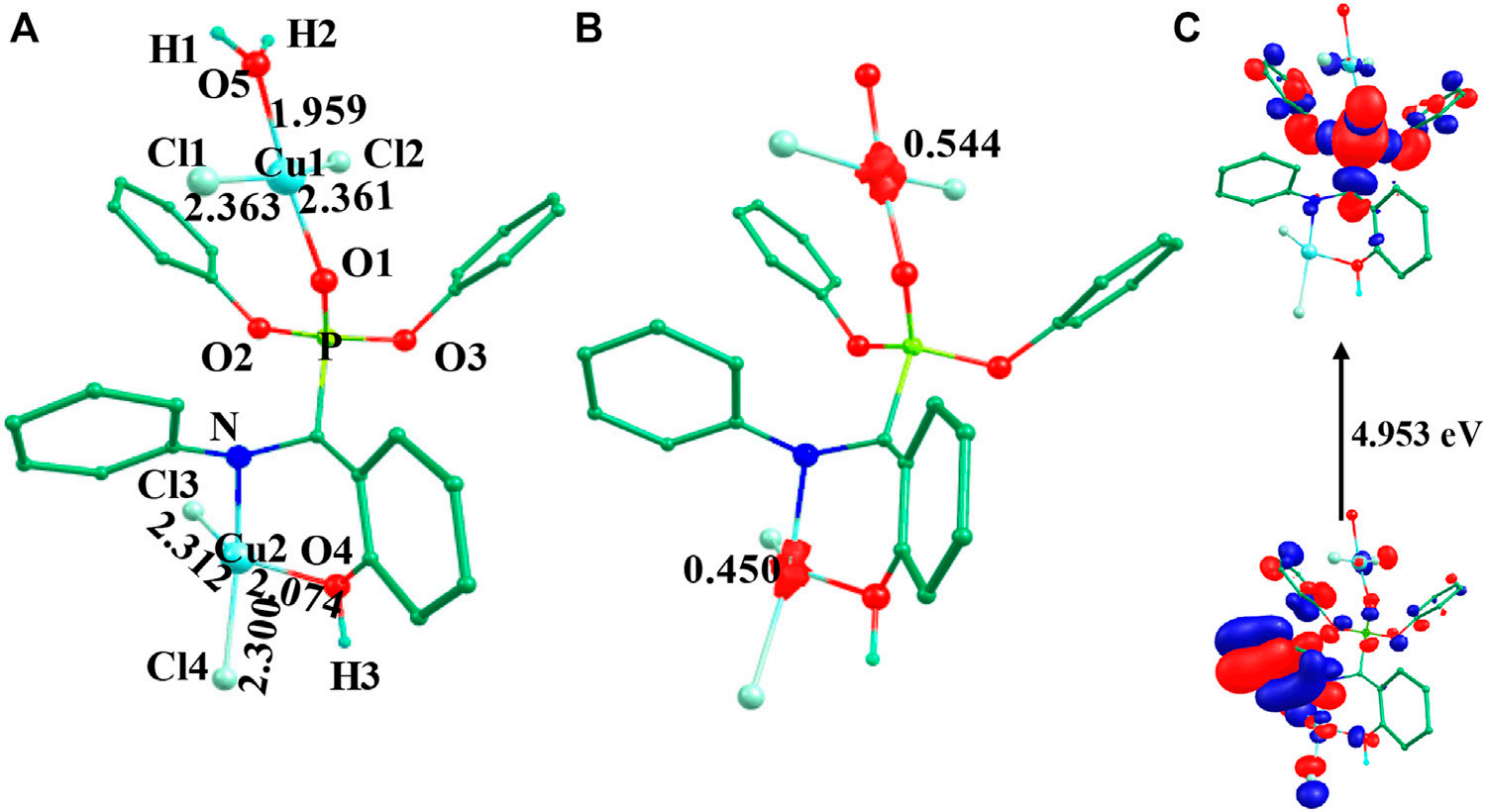
B3LYP-D2 **(A)** optimized structure, **(B)** spin density plot and **(C)** HOMO-LUMO gap of the species ^3^ **IV** (Bond lengths are in Å).

Here, species **IV** is the dinuclear derivative of species **I** where halogen and water substituents are coordinated to the metal centre. Species **IV** has previously been well characterized by analytical, spectral and thermal data (Azzam et al., 2020). For this species, two spin states ^3^ **IV** and ^1^ **IV** are possible due to ferro and antiferromagnetic interactions between the metal centres (see Table 1). Following optimization of both spin states, our computed data revealed ^3^ **IV** as the ground state with ^**1**^ **IV** lying 2.5 kJ/mol high in energy (see Figure 2 and Table 1). The magnetic exchange *J* value was calculated as 23.9 cm^−1^, in accordance with experimental data (Azzam et al., 2020).

**TABLE 1:** Possible spin states of species **IV** and **V**.

Electronic configuration			
Spin state	Cu(II)	Cu(II)	Relative energy (kJ/mol)
[(Cu^II^)_2_(L)(Cl)_4_(H_2_O)]			
^3^IV	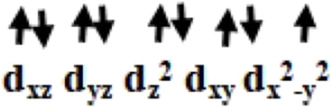	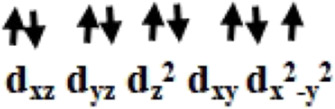	0.0
^1^IV	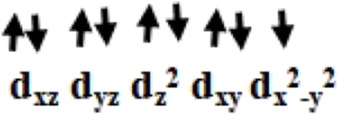	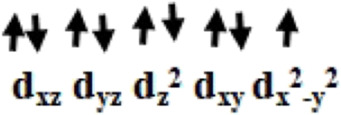	2.5
[(Cu^II^)_2_(L)(Cl)_2_(Br)_2_(H_2_O)]			
^3^V	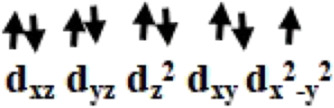	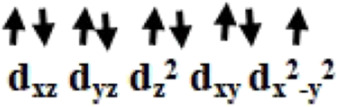	0.0
^1^V	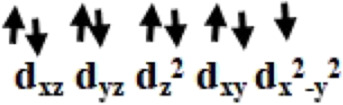	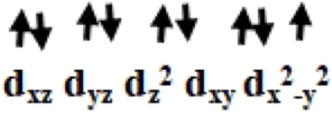	2.6

The optimized geometry and spin density plot for ^3^ **IV** are shown in Figure 2. The computed Cu1-Cl_avg_ and Cu2-Cl_avg_ bond lengths were found to be 2.362 Å and 2.306 Å, respectively. Similarly, Cu1-O5, Cu1-O1, Cu2-N and Cu2-O4 bond lengths were computed to be 1.959 Å, 1.945 Å, 2.117 Å and 2.074 Å, respectively. Similar to species **I**-**III**, both copper centres adopted a distorted square planar geometry towards tetrahedral which was evident from the bond angles 173.2°, 87.1°, 148.9°, 104.9° and 160.2° for ∠Cl1-Cu1-Cl2, ∠Cl1-Cu1-O5, ∠Cl3-Cu2-O4, ∠Cl3-Cu2-Cl4, ∠N-Cu2-Cl4, respectively. The two phenyl rings of the phosphonate moiety were coplanar to each other and the remaining two moved out of the plane to reduce strain in the system. The Cu1 and Cu2 centres had a spin density value of ρ = 0.544 and ρ = 0.450, respectively, corresponding to the presence of unpaired electron at both the metal centres (see Figure 2B). The HOMO-LUMO gap was computed to be 4.953 eV and was higher than those computed for species **I**-**III**. The electronic configuration at both copper centres was found to be (d_xz_)^2^ (d_yz_)^2^ (d_xy_)^2^ (d_z_^2^)^2^ (d_x_^2^_-y_^2^)^1^ (see Supplementary Figure S5). The optimized structure, spin density plot and HOMO-LUMO gap of ^1^ **IV** is shown in Supplementary Figure S6.

A DFT study was carried out on species **V** (model species of **IV)** where the chloride ligand at Cu2 was replaced by the bromide ligand. Following optimization of both possible spin interactions, our results revealed that ^3^ **V** was computed to be the ground state with ^1^ **V** lying 2.6 kJ/mol high in energy (see Table 1). The magnetic exchange *J* value was calculated to be 11.5 cm^−1^ which suggested the ferromagnetic nature of this species (Ansari et al., 2017; Azzam et al., 2020; Yadav et al., 2021).

**FIGURE 3:**
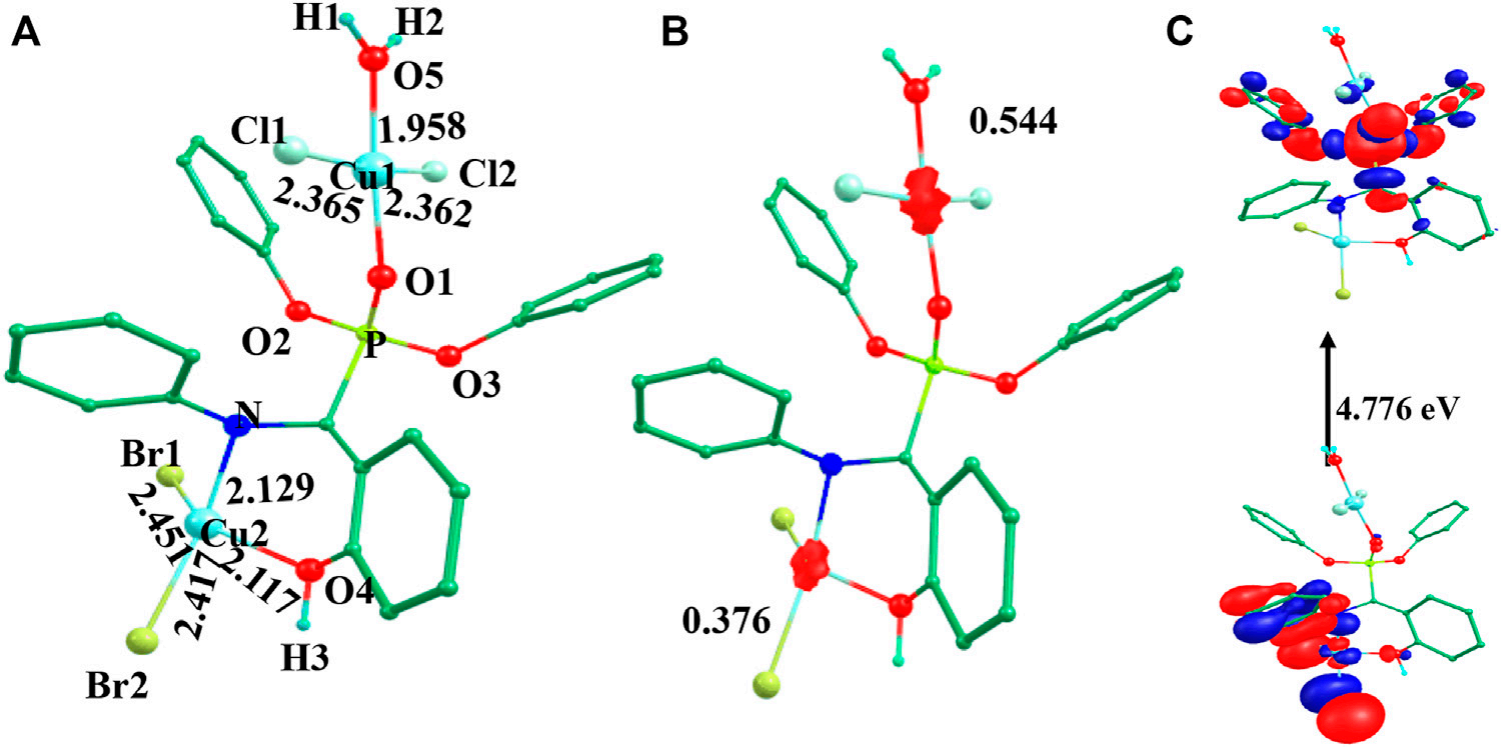
B3LYP-D2 **(A)** optimized structure, **(B)** spin density plot and **(C)** HOMO-LUMO gap of the species ^3^ **V** (Bond lengths are in Å).

The optimized geometry and spin density plot of ^3^ **V** are shown in Figure 3. The computed Cu1-Cl_avg_ and Cu2-Br_avg_ bond lengths were found to be 2.363 Å and 2.434 Å, respectively. The main structural parameters including bond lengths Cu1-O5, Cu1-O1, Cu2-N and Cu2-O4 were computed to be 1.958 Å, 1.945 Å, 2.129 Å and 2.117 Å, respectively. A distorted square planar geometry towards tetrahedral was predicted at both the copper centres which was apparent from the bond angle values of 103.8°, 141.4° and 157.9° for ∠Br1-Cu2-Br2, ∠Br1-Cu2-O4, ∠Br2-Cu2-N, respectively. The distortion in geometry was more pronounced at Cu2 compared to Cu1. The spin density plot suggested that more delocalization was favored at the Cu2 centre (*ρ* = 0.544(Cu1)/0.376(Cu2); see Figure 3B. The electronic configuration at both copper centres was found to be (d_xz_)^2^ (d_yz_)^2^ (d_xy_)^2^ (d_z_^2^)^2^ (d_x_^2^_-y_^2^)^1^. The HOMO-LUMO gap of ^3^ **V** was computed to be 4.776 eV. The optimized structure, spin density plot and HOMO-LUMO gap of ^1^ **V** is shown in Supplementary Figure S9.

### Molecular electrostatic potential maps

**FIGURE 4:**
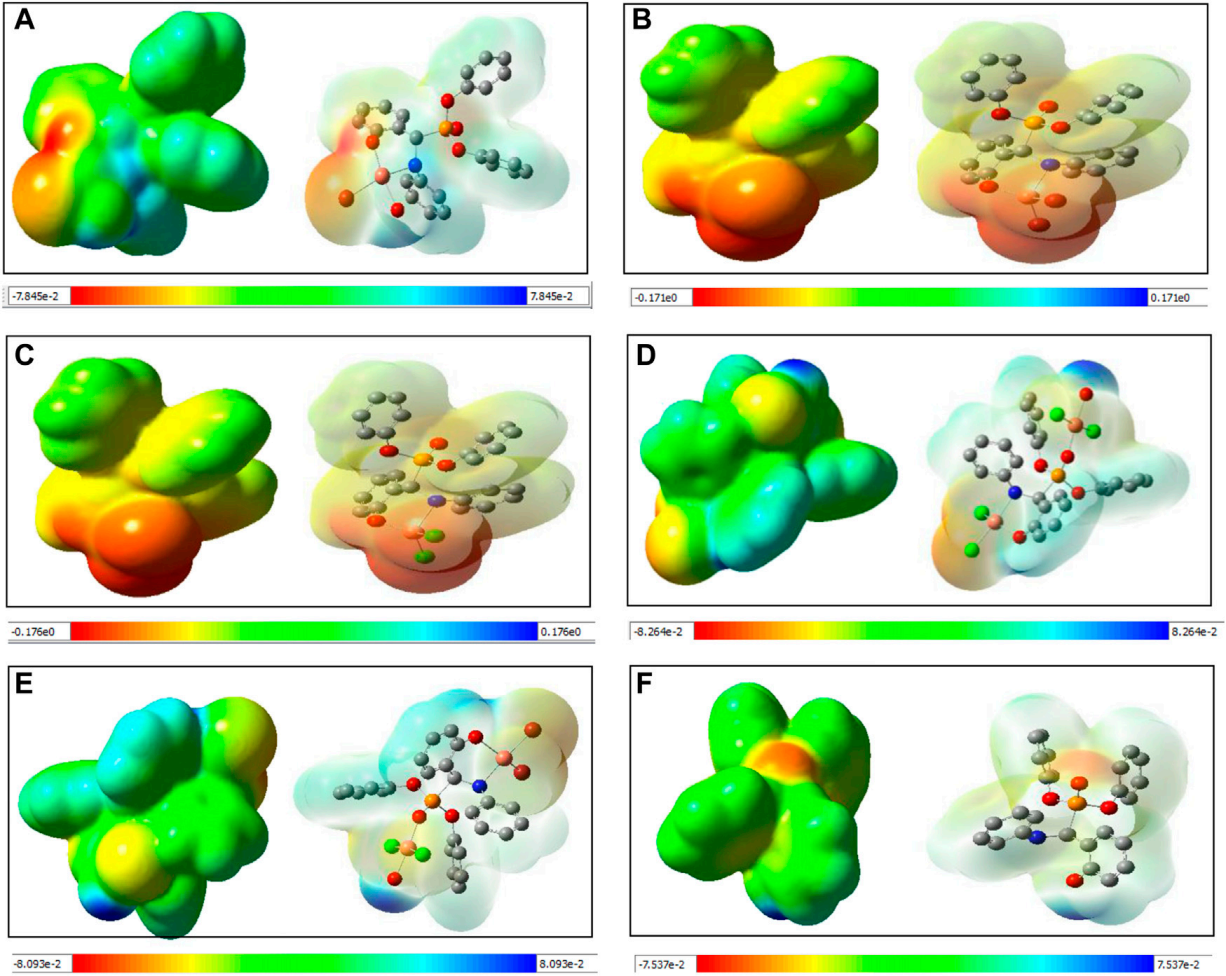
Computed molecular electrostatic potential maps (solid/transparent) of **(A)** species **I**, **(B)** species **II**, **(C)** species **III**, **(D)** species **IV**, **(E)** species **V**, and **(F)** Ligand.

To exploit the positive and negative potential regions, molecular electrostatic surfaces were mapped for all five species. Different regions of electrostatic potential are commonly described with a colour grading, using red for the most negative potential surface, blue for the most positive potential, and green and yellow for a potential between the two extremes (Drissi et al., 2015). The electrostatic potential maps of species **I** to **V** are shown in Figure 4. The electrostatic potential distribution for the molecule in the crystal was calculated from Eq. 1 (Takayanagi et al., 1996):

$$\Phi \left(r\right)=\int \frac{\rho \left(r\right)}{\left|r-r^{\prime }\right|}dr$$ (1)

A close inspection of the MEP maps revealed that the presence of localized negative potential regions were located at the O atom of the phosphonate moiety in the mononuclear species **I**-**III** while the negative potential was dispersed to neighbouring atoms in both dinuclear species (**IV** and **V**). Within the mononuclear species, a more negative potential was located on species **II** and **III** compared to species **I**. Thus, it may be predicted that the model species **II** and **III** are more prone to electrophilic attack. It is also noteworthy that the region around the aromatic ring witnessed a gradual depletion in the red and blue colours and an increase in green. On the other hand, dinuclear species showed blue and green regions that possessed positive potentials.

### Natural bond analysis

**FIGURE 5:**
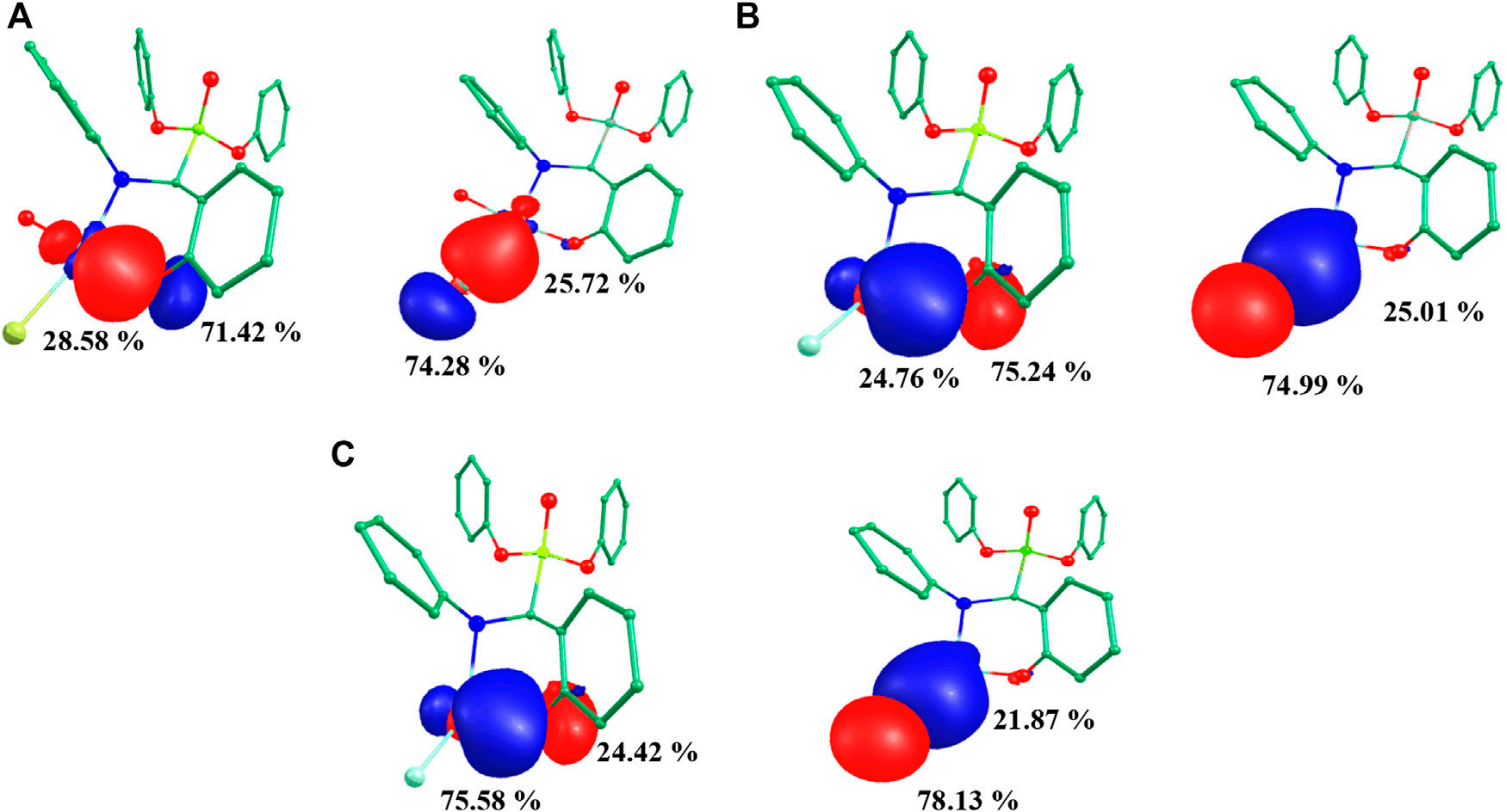
Computed NBO plots of **(A)** species **I**, **(B)** species **II** and **(C)** species **III**.

NBO analysis of all species was performed to gain in depth knowledge about the bonding aspects and charge transfer occurring in all five species. The NBO approach makes use of a set of localized bond, anti-bond and Rydberg extra valence orbitals. NBO analysis provides the representations of orbitals and the highest viable percentage of electron density based on mathematically accurate results (Monika et al., 2021). All five species showed a common backbone skeleton comprising of *α*-aminophosphoante ligated to the metal core. The only differences were in the substituents present at the metal centre. Thus, the study of the interaction of substituents with the copper centre helped to better understand the bonding aspects of all species under investigation. The NBO plots of substituents with the metal of species **I**-**III** are shown in Figure 5. The examination of the NBO plot of species **I** revealed that the Cu-O5 bond was quite polar in nature with 28.58% orbital contribution from copper and 71.42% from oxygen (O5). Similarly, the orbital contributions of the Cu-Br bond was computed to be 74.28% by bromine and 25.72% by copper metal. The NBO plots were also computed for the model species **II** and **III**. Among the mononuclear species, the metal-substituent bond was more polar in the case of model species **III**. In the dinuclear species ^3^ **IV**, the NBO plots were computed for both metal centres. The orbital contribution for the Cu1-Cl1 bond was computed to be 23.10% by Cu1 and 76.90% by Cl1. The orbital contribution for the highly polar Cu1-Cl2 bond was computed to be 21.94% by Cu1 and 78.06% by Cl2. The orbital contribution for the Cu2-Cl3 bond was computed to be 29.47% by Cu2 and 70.53% by Cl3. The orbital contribution for the Cu2-Cl4 bond was computed to be 27.04% by Cu2 and 72.96% by Cl4. Similarly, Cu-Cl_avg_ orbital contribution for ^3^ **IV** was computed to be 22.5% by Cu1 and 77.5% by chloride. Cu-Br_avg_ orbital contribution of ^3^ **IV** was computed to be 32.81% by Cu2 and 67.19% by bromide (Supplementary Figure S11). The NBO plots for ^1^ **IV** and ^1^ **V** are shown in Supplementary Figure S12.

**FIGURE 6:**
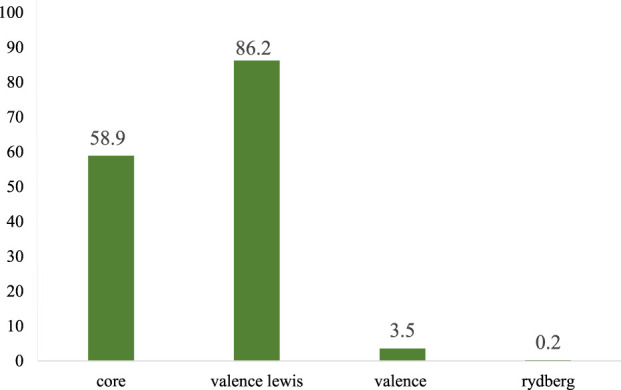
Bar diagram representing the natural population analysis of species **I**.

The structure of species **I** exhibited a type of Lewis structure (99.70%), 0.18% of valence non-Lewis, and 0.10% of Rydberg non-Lewis (Figure 6). Both Species **II** and **III** exhibited a type of Lewis structure (99.99%) and a negligible percentage of valence non-Lewis and of Rydberg non-Lewis. The data for the remaining are shown Supplementary Figures S13–18.

### Perturbation theory

NBO analysis allows to explore donor-acceptor interactions by making use of the second-order perturbation approach (Fock matrix). The strength of interaction stabilization energy E^(2)^ related with electron delocalisation among the donor (i) and acceptor (j) was calculated using the following equation:

$$E^{\left(2\right)}=q_{i\;\frac{F^{2}\left(i,j\right)}{\varepsilon_{j-\varepsilon_{i}}}}$$ (2)

where q_i_ is the donor orbital occupancy, *ε*_j_ and *ε*_i_ depict diagonal elements and F (i, j) denotes off diagonal NBO Fock matrix element. The more intensive the interaction between the acceptor-donor electron, the greater the value for stabilization energy *E* ^*(2)*^ is*,* indicative of greater conjugation in the whole system (Sumrra et al., 2018). The feasible dominant intensive interactions of species **I** are listed in Table 2. The computed data showed that strong intramolecular hyper-conjugative interactions stabilized by pi electrons were present in species **I**. The system was mainly stabilized by hyper-conjugative interactions of π(C31-C34) → π*(C29-C30), π(C29-C30) → π*(C32-C36), π(C32-C36) → π*(C31-C34), π(C44-C47) → π*(C42-C43) responsible for conjugation in the aromatic ring (see Supplementary Figure S19). Further conjugation was due to interactions π(C45-C49) → π*(C44-C47), π (C6-C10) → π*(C5-C8), π(C6-C10) → π*(C3-C4), π(C5-C8) → π*(C3-C4). The most important (n → π*) interactions were n (C19) → π*(C15-C17), n (C19) → π*(C16-C18), and n (O28) → π*(C29-C30) with a stabilization energy of 33.79, 28.99 and 7.75 kcal/mol, respectively. Further stabilization was achieved by interactions n (O26) → σ*(P25-O28) and n (O26) → σ*(P25-O27) having a stabilization energy of 9.39 and 12.40 kcal/mol, respectively. A similar trend was computed in the model species **II** (see Supplementary Table S2 and Supplementary Figure S20) and **III** (see Supplementary Table S3 and Supplementary Figure S21).

**TABLE 2:** Perturbation theory energy analysis of species **I**.

Donor NBO (i)	Acceptor NBO (i)	E^(2a)^kcal/mol	E(j)-E(i)^b^(a.u.)	F(i,j)^c^(a.u.)
π (C3–C4)	π *(C5–C8)	10.04	0.28	0.067
π (C3–C4)	π *(C6–C10)	9.19	0.29	0.065
π (C5–C8)	π *(C3–C4)	10.54	0.27	0.068
π (C5–C8)	π *(C6–C10)	9.27	0.28	0.065
π (C6–C10)	π *(C3–C4)	10.72	0.26	0.067
π (C6–C10)	π *(C5–C8)	10.87	0.26	0.068
π (C29–C30)	π *(C31–C34)	9.26	0.28	0.065
π (C29–C30)	π *(C32–C36)	10.22	0.28	0.068
π (C31–C34)	π *(C29–C30)	11.34	0.27	0.070
π (C31–C34)	π *(C32–C36)	9.37	0.27	0.064
π (C32–C36)	π *(C29–C30)	9.53	0.26	0.064
π (C32–C36)	π *(C31–C34)	10.74	0.27	0.068
π (C42–C43)	π *(C44–C47)	9.39	0.28	0.065
π (C42–C43)	π *(C45–C49)	9.93	0.28	0.067
π (C44–C47)	π *(C42–C43)	11.16	0.27	0.069
π (C44–C47)	π *(C45–C49)	9.55	0.27	0.064
π (C45-C49)	π *(C42–C43)	9.72	0.26	0.065
π (C45–C49)	π *(C44–C47)	10.54	0.27	0.067
n (O1)	n*(Cu53)	6.70	0.75	0.090
n (N2)	n*(Cu53)	18.33	0.70	0.148
n (O26)	σ*(P25-O 28)	9.39	0.37	0.075
n (O26)	σ*(P25-O 27)	12.40	0.36	0.085
n (O28)	π *(C29-C30)	7.75	0.37	0.072

In the dinuclear species ^3^ **IV**, the system was mainly stabilized by π→ π* transitions, including π(C6-C10) → π*(C3-C4), π(C6-C10) → π*(C5-C8), π(C16-C18) → π*(C14-C15), π(C17-C19) → π*(C16-C18), π(C31-C34) → π*(C29-C30), π(C44-C47) → π*(C42-C43), π(C45-C49) → π*(C44-C47) (see Supplementary Table S4 and Supplementary Figure S22) with respective stabilization energies of 10.53, 10.01, 12.22, 11.29, 11.17, 11.13 and 10.71 kcal/mol (see Supplementary Table S4 for remaining transitions). Other important intra-molecular hyper-conjugative interactions were also present, providing extra stability to the system. These interactions included n → n* transitions such as n (O1) → n*(Cu53) with a stabilization energy 9.95 kcal/mol and n (N2) → n*(Cu53) with a stabilization energy of 14.76 kcal/mol. The dominant donor-acceptor interactions of ^1^ **IV** are shown in Supplementary Table S5 and Supplementary Figure S22. The Fock-matrix based second order perturbation theory transitions for the remaining species (^3^ **IV** and ^1^ **IV**) are listed in Supplementary Tables S6,7 and Supplementary Figure S23.

### Natural population analysis charges

The computation of NPA charges helps to predict the reactivity of a system by analysing the presence of electrophilic and nucleophilic centres (Biswas et al., 2021). The NPA charges of heteroatoms of all five species are listed in Table 3. The NPA charges were computed as −0.506 (O1) and −0.246 (O4) for species **I**, −0.516(O1), −0.268(O4), −0.217(Cl1), −0.277 (Cl2) for species **II** and −0.517 (O1), −0.268(O4), −0.248 (Br1), −0.304 (Br2) for species **II**. Among the mononuclear species, the NPA charges were more negative in the case of model species (**II** and **III**) compared to previously reported species (species **I**). Among the model species, species **III** was computed to have more negative NPA charges, thus predicting its comparatively higher nucleophilic character (see Table 3). In the case of dinuclear species, the computed NPA charges were −0.499 (O1), −0.324 (O4), −0.236(Cl1), −0.221 (Cl2), −0.169 (Cl3), −0.190 (Cl4) for species ^3^ **IV** and −0.499 (O1), −0.327 (O4), −0.236 (Cl1), −0.221 (Cl2), −0.121 (Br1), −0.141 (Br2) for species ^1^ **V**. These NPA charges were comparable for both species except for the halogen atoms which were more negative for species **IV**. The NPA charge analysis predicted that the heteroatoms of the mononuclear species were more nucleophilic than those of the dinuclear species. Among the mononuclear species, the heteroatoms of the model species **III** were predicted to be more nucleophilic.

**TABLE 3:** Computed NPA charges of all the reported species.

	NPA charges					
Species	O1	O4	X1	X2	X3	X4
**I**	−0.506	−0.246	−0.207	—	—	—
**II**	−0.516	−0.268	−0.217	−0.277	—	—
**III**	−0.517	−0.268	−0.248	−0.304	—	—
^3^ **IV**	−0.499	−0.324	−0.236	−0.221	−0.169	−0.190
^3^ **V**	−0.499	−0.327	−0.236	−0.221	−0.121	−0.141

Where X1 = -Br for species **I**; X1, X2 = -Br for species **II**; X1, X2 = -Cl for species **III**; X1, X2, X3, X4 = -Cl for species **IV**; X1, X2 = Cl and X3, X4 = -Br for species **V**.

### Molecular docking study

Several *α*-aminophosphonate derivatives, exhibiting interesting biological properties, have previously been synthesized (El-Boraey et al., 2015; Gundluru et al., 2021; Tian et al., 2022). Many studies have reported their potential as antiviral agents (Hu et al., 2008; Song et al., 2010; Amira et al., 2021). Here we performed a molecular docking study of ligand L and species **I**-**V** against the target proteases PDB ID: 6LU7 (SARS-CoV-2) and PDB ID: 7T9K (SARS-CoV-2 Omicron). The high-resolution 3D structures of both SARS-CoV-2 proteases were downloaded from the protein data bank (https://www.rcsb.org/). Docking calculations were carried out using the AutoDock Vina software and AutoDockTools (ADT) (Trott et al., 2010). Prior to the docking calculations, all water molecules were removed. It was ensured that the residues of the active site of each protein were included when assigning both grid box sizes (30 × 30 × 30 Å centered at X = -10.773, Y = 12.066, Z = 68.545 for 6LU7 and 27 × 27 × 27 Å centered at X = 178.412393, Y = 147.822071 and Z = 222.093571 for 7T9K). All ligands were geometrically optimized geometries and the docked poses and ligand-protein interactions were visualized using the Discover Studio Visualizer 4.0 software (BIOVIA Systemes, Dassault, 2021). The analysis of the docked models was performed to investigate the binding affinity and the nature of the intermolecular bonding interactions between each species and the target proteins. The modes of interactions of the ligand with proteins were determined by investigating their favourable orientations of binding.

**FIGURE 7:**
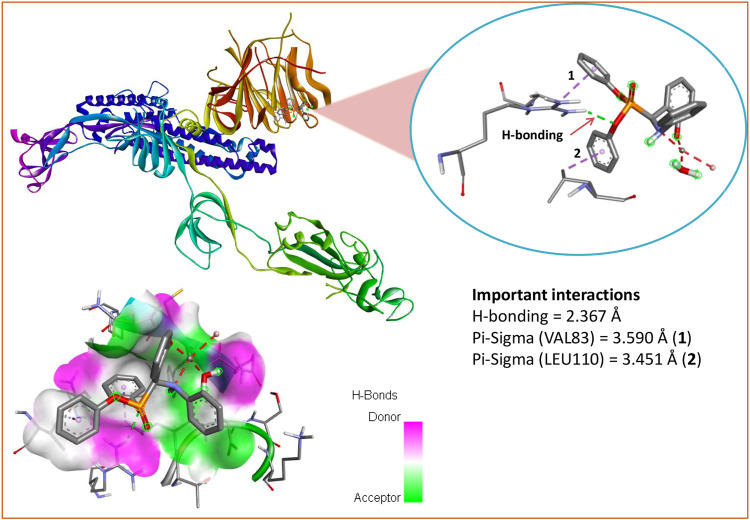
Molecular docking study of species **I** with the SARS-CoV-2 Omicron variant protease (7T9K).

The molecular docking of all species (**I**-**V**) with both SARS-CoV-2 proteases revealed that the mononuclear species (**I**-**III**) had more binding affinity than the dinuclear species (**IV**-**V**) for the protein targets (see Table 4). Among all species, species **I** had the highest binding affinity (binding energy -5.9 kcal/mol) with the SARS-CoV-2 Omicron protease. The favourable orientation and important interactions of species **I** and the Omicron protease are shown in Figure 7. Species **I** showed two types of significant interactions, one was H-bonding and the other was Pi-Sigma interactions. H-bonding occurred at a distance of 2.367 Å and Pi-Sigma interactions with VAL83 and LEU110 occurring at a distance of 3.590 Å and 3.451 Å, respectively. The molecular docking results obtained for the remaining species are shown in Supplementary Figures S24–S34.

**TABLE 4:** Favourable binding energies (docking scores) for the docked poses for species **I-V** with the selected SARS-CoV-2 proteases.

Species	Binding energy (kcal/mol)	
	6LU7	7T9K
**I**	−5.6	−5.9
**II**	−5.8	−5.4
**III**	−5.8	−5.3
^3^ **IV**	−4.5	−4.9
^1^ **IV**	−5.1	−5.2
^3^ **V**	−5.1	−5.3
^1^ **V**	−5.4	−5.3

### Comparative study

Species **I**-**III** are mononuclear whereas species **IV**-**V** are dinuclear. Species **II**, **III** and **V** are model species. All species showed a distortion in geometry from square planar towards tetrahedral. Among the mononuclear species, **I** was computed to have the highest HOMO-LUMO gap. In the case of the dinuclear species, low spin states had a comparatively lower HOMO-LUMO gap compared to high spin states. The computed MEP maps predicted that the mononuclear species had more negative potential, and thus may show be more electrophilic than the dinuclear species which had a comparatively more positive potential. Further theoretical reactivity predictions based on MEP analysis suggested that the model species **II** and **III** had the most electrophilic regions. NPA charge analysis predicted that the heteroatoms of the mononuclear species were more nucleophilic than those of the dinuclear species and among them, the heteroatoms of species **III** were more nucleophilic. NBO analysis suggested that all systems were stabilized by intramolecular hyper-conjugative interactions. The stabilization energy value (E^(2a)^) values indicated that the dinuclear species E^(2a)^ were comparatively more stabilized with these interactions compared to the mononuclear species. Among the mononuclear species, the model species gained more stabilization as evident from their higher values of (E^(2a)^). Molecular docking studies indicated that all species showed interactions with various amino acid residues of two SARS-CoV-2 proteases. The mononuclear species were predicted to exhibit a higher binding affinity for the protein targets compared to the parent ligand as evident by their higher docking scores and number of hydrogen and hydrophobic bonds with the targets.

## Conclusion

Cu^II^ *α*-aminophosphonate complexes have gained considerable interest due to their valuable biological properties. Here, we have carried out calculations on five species using DFT method using a dispersion corrected functional B3LYP-D2 method to predict the correct ground state of the dinuclear species; high spin (*S* = 3) for species **IV** and species **V**. Spin density values confirmed the presence of unpaired electron nature at the copper centre of species **I**, **II** and **III** and also on each copper centre of dinuclear species. Species ^1^ **V** was computed to have the smallest transient energy gap (2.233 eV) whereas species ^3^ **IV** was computed to have the highest energy gap (4.953 eV). Computed MEP maps predicted that the mononuclear species were more electrophilic than the dinuclear species. Among the mononuclear species, the most negative potential was located on model species **II** and **III**. This was also evident from NPA charges analysis which suggested more negative charges present at heteroatoms in species **II** and **III**. Among these two, NPA charges were found to be more negative for species **III**. NBO analysis suggested that all species were stabilized by hyper conjugative interactions. Molecular docking predicted that the metal complexed ligands showed better affinity for the SARS-CoV-2 targets than the parent ligand in agreement with experimental findings. Among the metal complexes, the mononuclear species have comparatively higher interactions than the dinuclear species. Further studies, including *in vitro* assays, are warranted to confirm the potential of Cu^II^ *α*-aminophosphonate complexes as new therapeutic agents against COVID-19.

## Data Availability

The original contributions presented in the study are included in the article/Supplementary Material, further inquiries can be directed to the corresponding authors.
