# Supplementary material for: Theoretical exploration on structures, bonding aspects and molecular docking of α-aminophosphonate ligated copper complexes against SARS-CoV-2 proteases
Source: Front Pharmacol. 2022 Oct 3;13:982484. doi: 10.3389/fphar.2022.982484 (PMC9575937; doi:10.3389/fphar.2022.982484)
Supplement: Supplementary file 1 [file DataSheet1.PDF]

Theoretical Exploration on Structures, Bonding Aspects and Molecular Docking of α-Aminophosphonate Ligated Copper Complexes against SARS-CoV-2 Proteases

Oval Yadav[a], Manjeet Kumar[a], Himanshi Mittal[a], Kiran Yadav[a], Veronique Seidel[b]* and Azaj Ansari[a]*

[a]*Department of Chemistry, Central University of Haryana, Mahendergarh-123031, India.*

[b]*Natural Products Research Laboratory, Strathclyde Institute of Pharmacy and Biomedical Sciences, University of Strathclyde, Glasgow G4 0RE, United Kingdom*

***Corresponding authors**

Email addresses: ajaz.alam2@gmail.com (Azaj Ansari) and veronique.seidel@strath.ac.uk (Veronique Seidel)

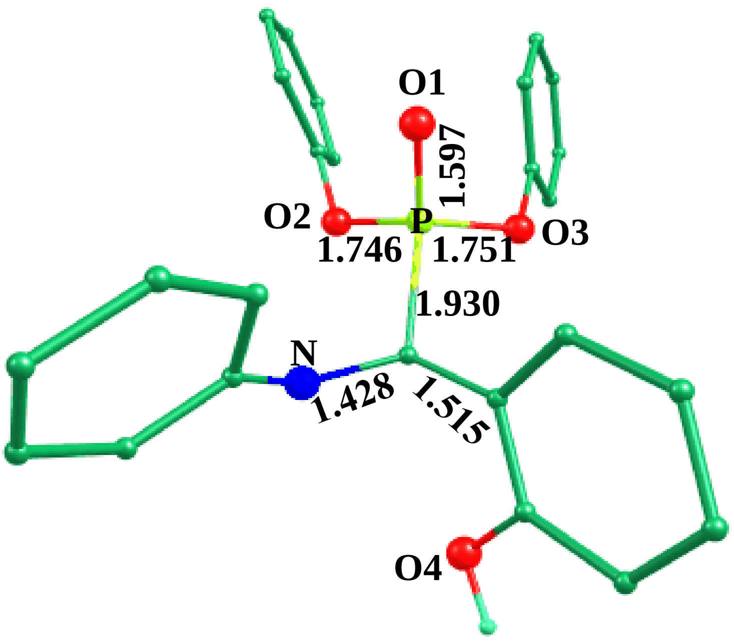

Fig. S1. B3LYP-D2 optimized structure of ligand L.

Table S1. Computed spin densities of species **I-V**.

**Species**	**Spin density**	
	**Cu1**	**Cu1**
**I**	0.408	-
**II**	0.427	-
**III**	0.470	-
^{3}**IV**	0.544	0.450
^{1}**IV**	0.543	0.449
^{3}**V**	0.544	0.376
^{1}**V**	-0.543	0.375

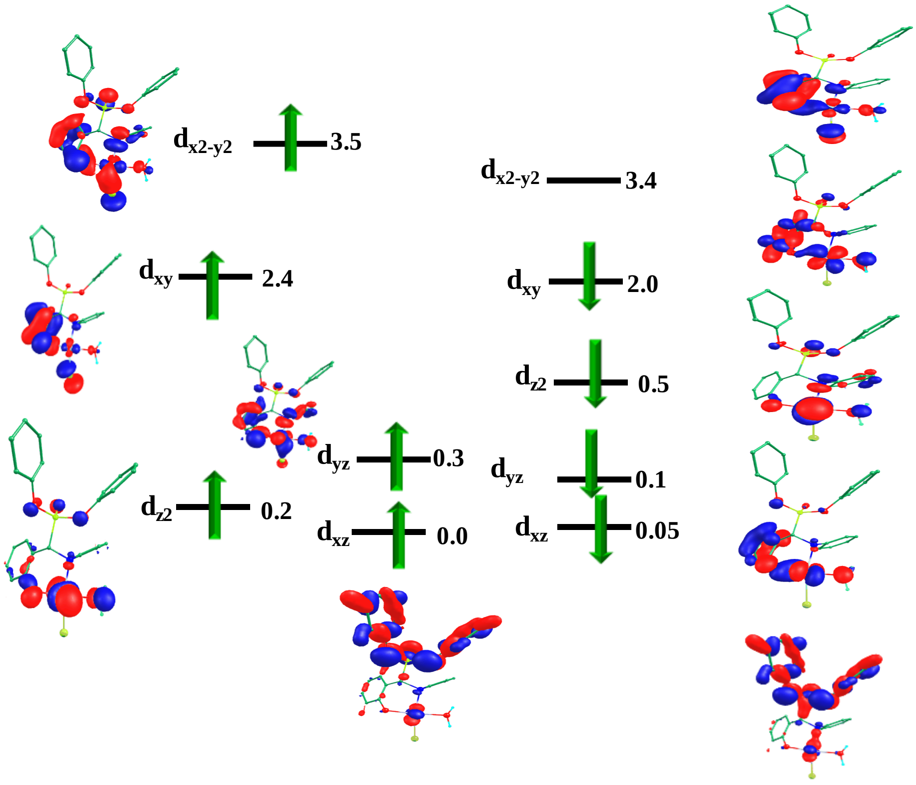

Fig. S2. Eigenvalue plot incorporating energies of d-based orbitals for alpha and beta spin corresponding species **I** (in eV).

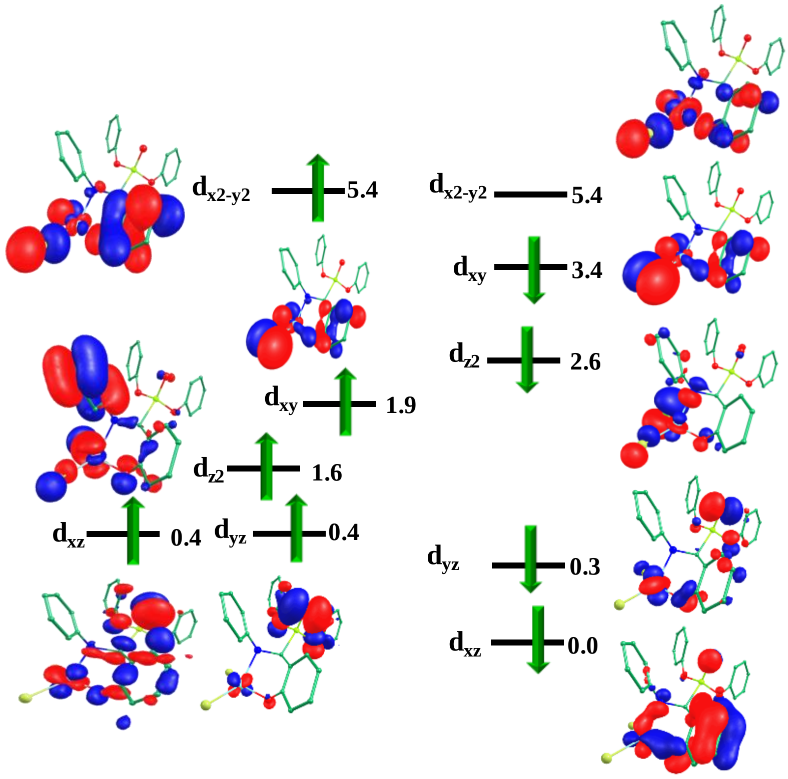

Fig. S3. Eigenvalue plot incorporating energies of d-based orbitals for alpha and beta spin corresponding species **II** (in eV).

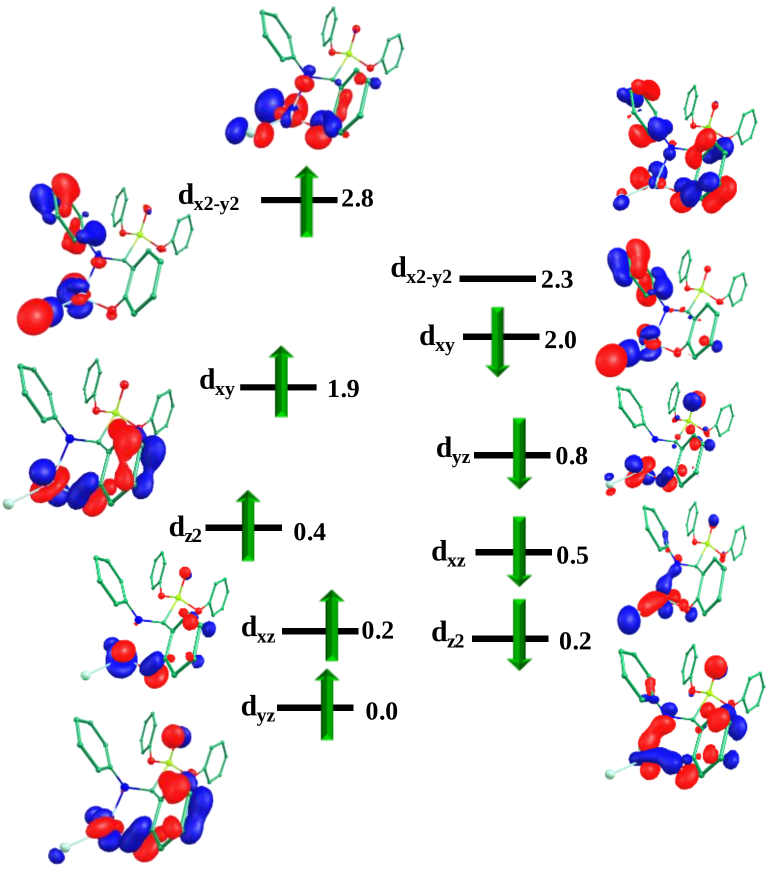

Fig. S4. Eigenvalue plot incorporating energies of d-based orbitals for alpha and beta spin corresponding species **III** (in eV).

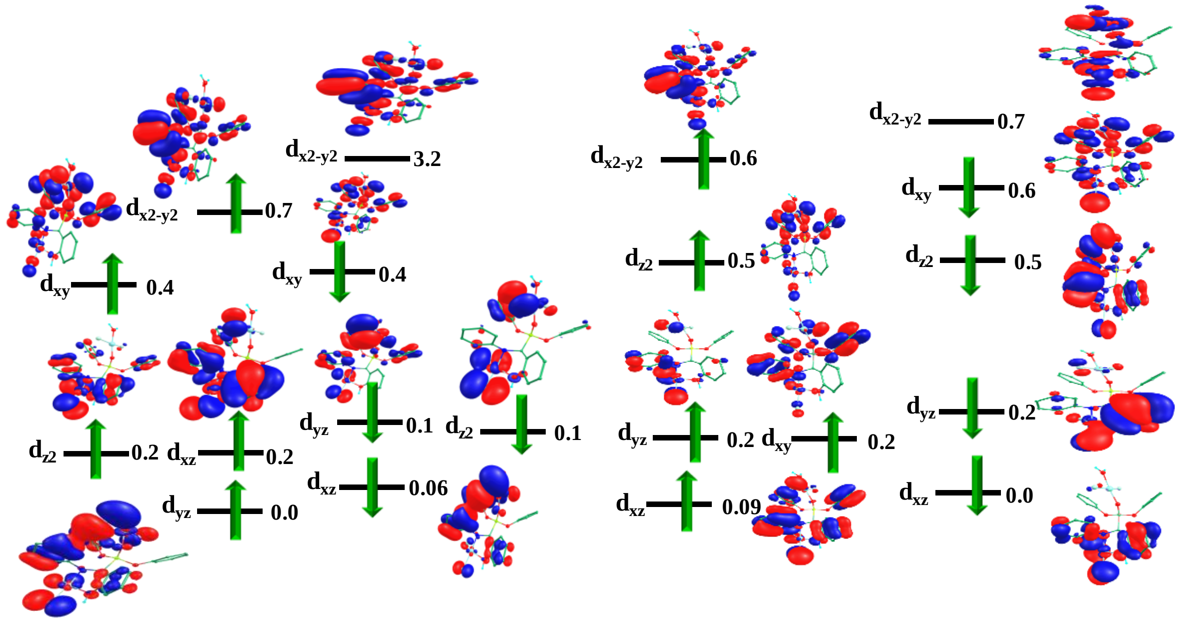

Fig. S5. Eigenvalue plot incorporating energies of d-based orbitals for alpha and beta spin corresponding species ^{3}**IV** (in eV).

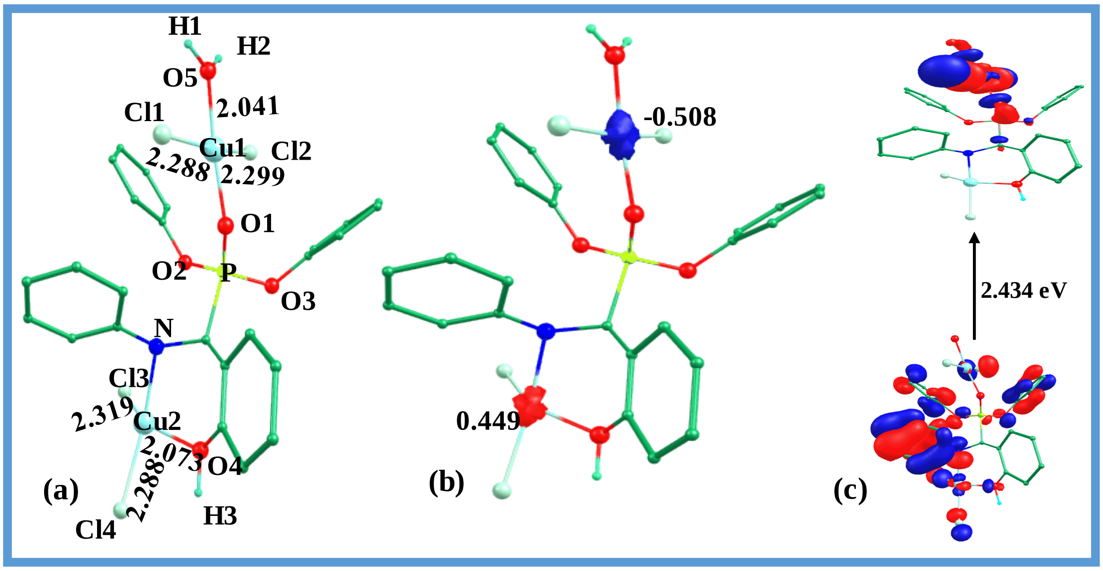

Fig. S6. B3LYP-D2 (a) optimized structure, (b) spin density plot and (c) HOMO-LUMO gap of the species [1]**IV**.

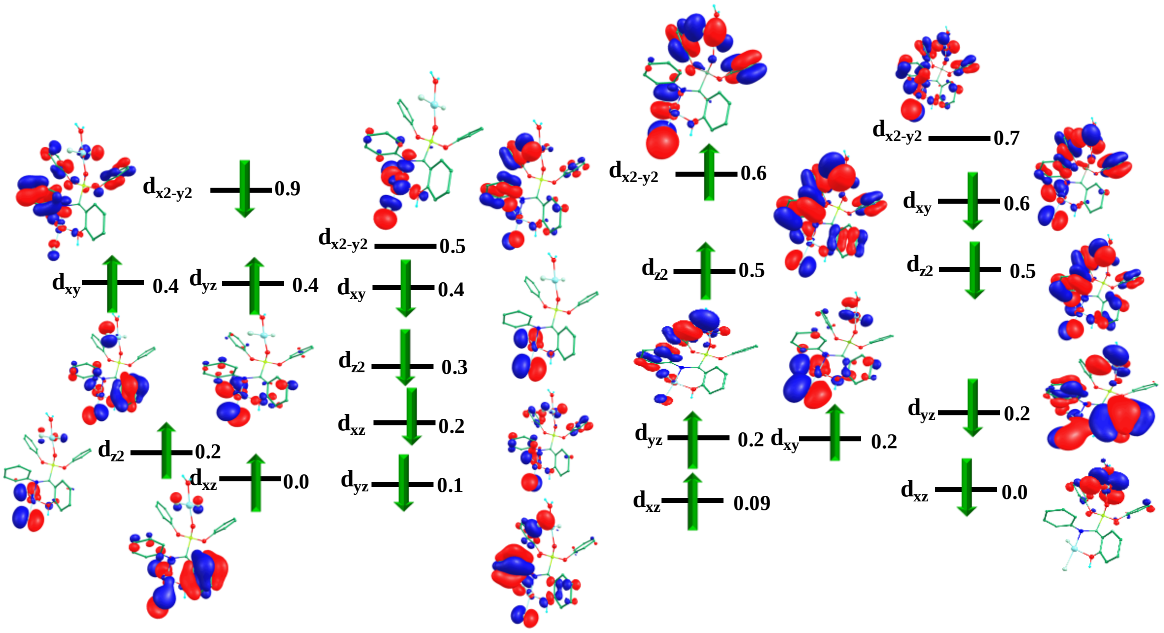

Fig. S7. Eigenvalue plot incorporating energies of d-based orbitals for alpha and beta spin corresponding species [1]**IV** (in eV).

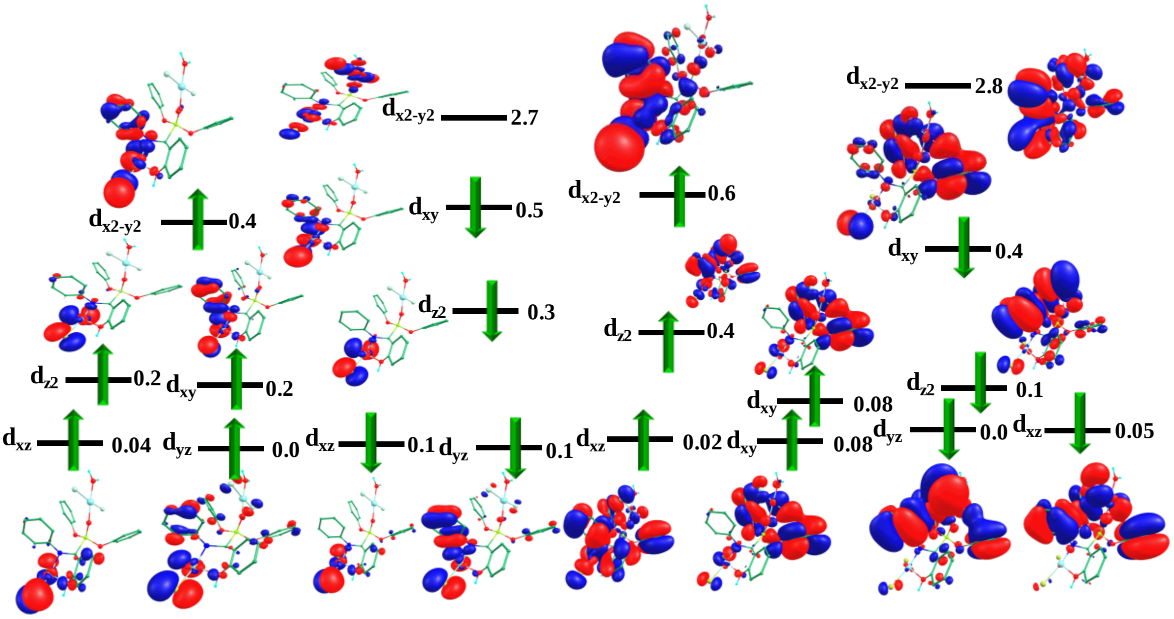

Fig. S8. Eigenvalue plot incorporating energies of d-based orbitals for alpha and beta spin corresponding species ^{3}**V** (in eV).

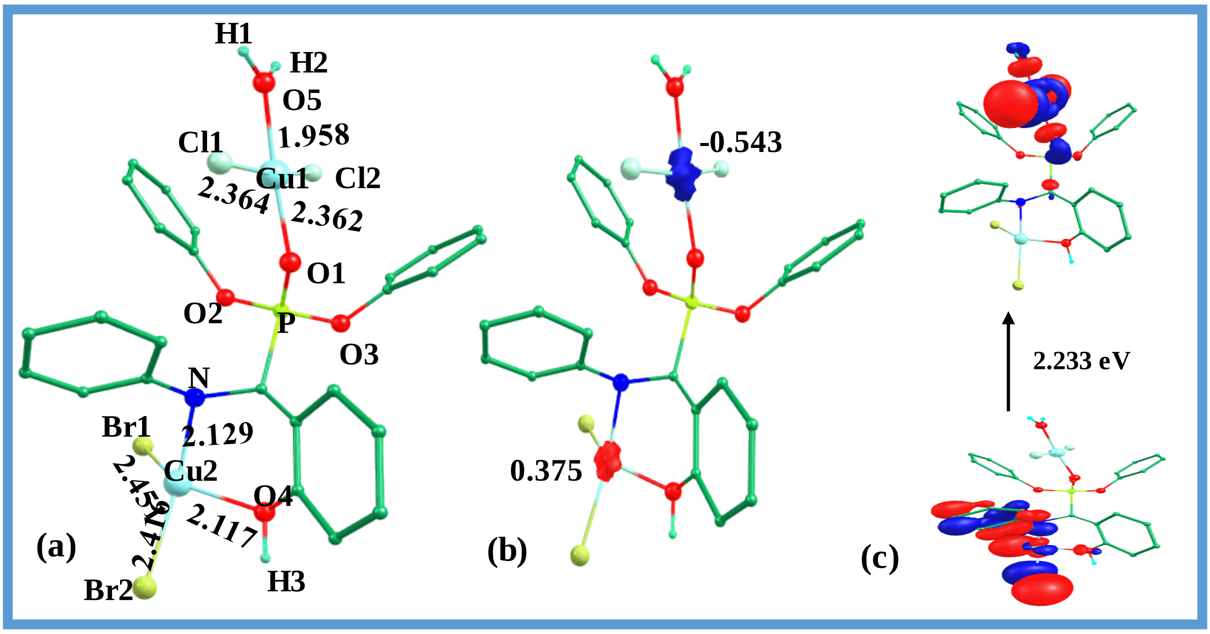

Fig. S9. B3LYP-D2 (a) optimized structure, (b) spin density plot and (c) HOMO-LUMO gap of the species ^{1}**V**.

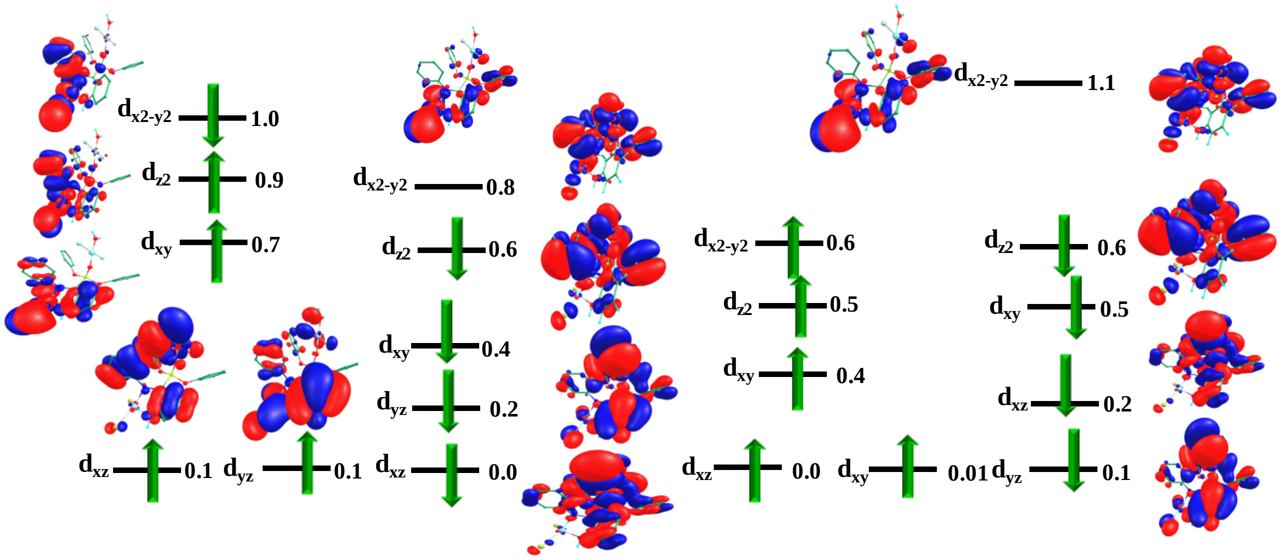

Fig. S10. Eigenvalue plot incorporating energies of d-based orbitals for alpha and beta spin corresponding species ^{1}**V** (in eV).

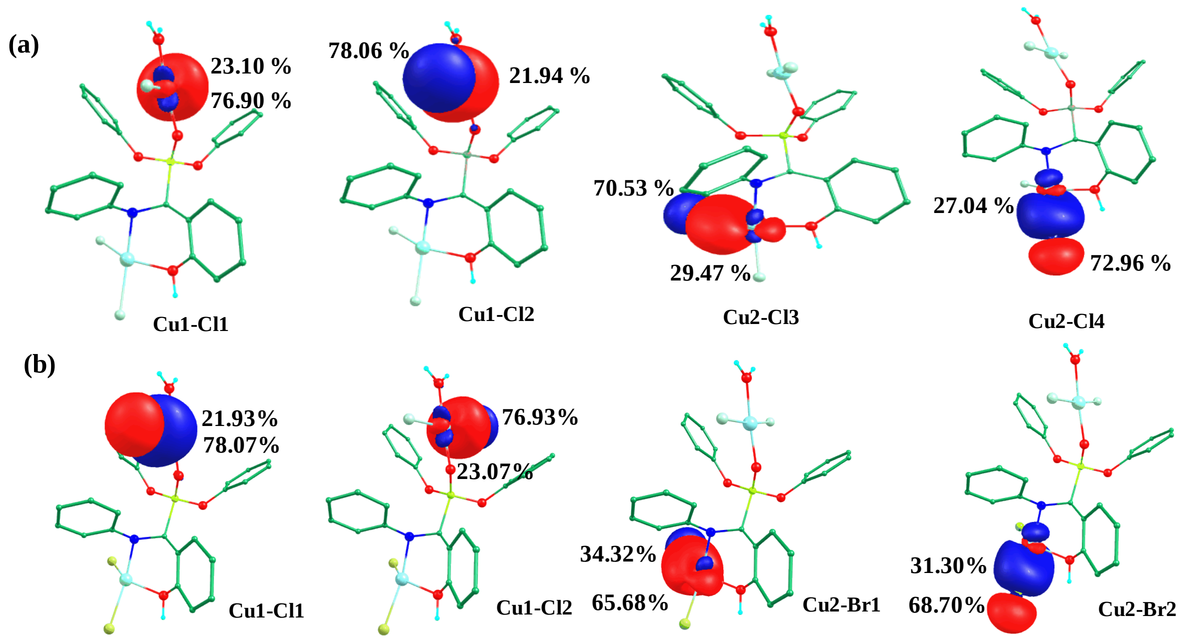

Fig. S11. Computed NBO plots for (a) ^{3}**IV** and (b) ^{3}**V**.

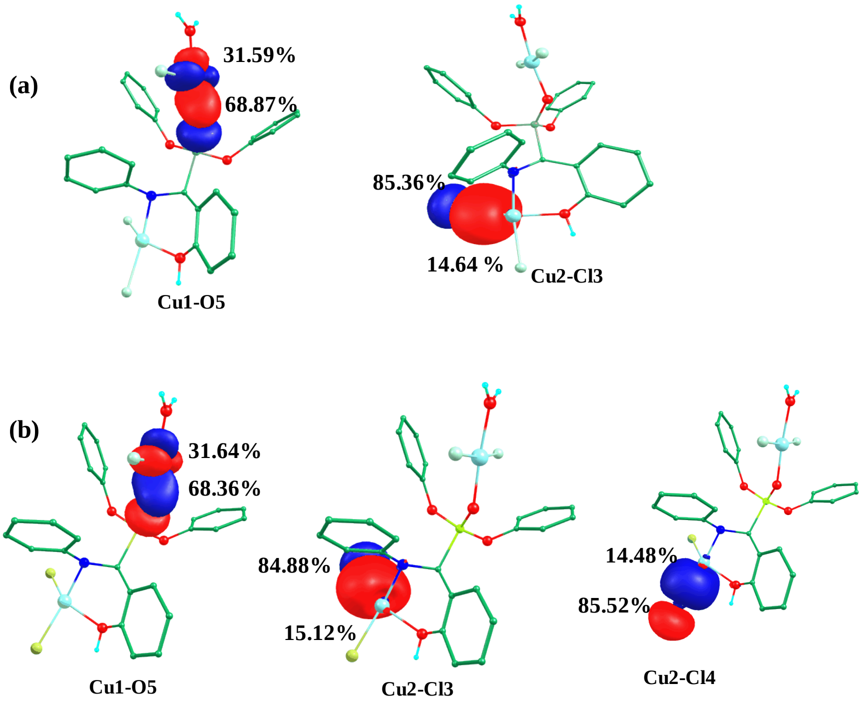

Fig. S12. Computed NBO plots for (a) [1]**IV** and (b) [1]**V**.

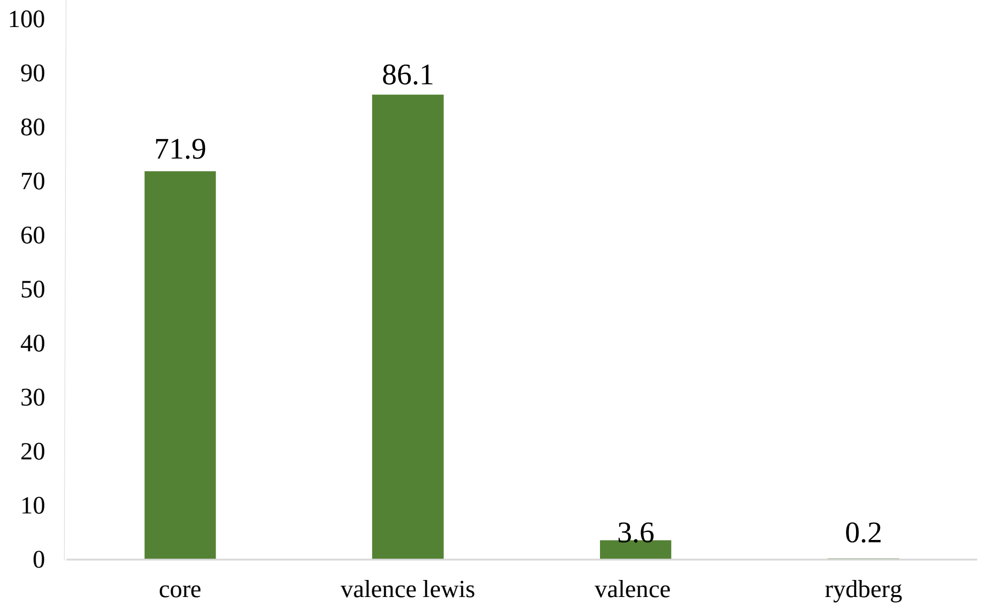

Fig.S13. Bar diagram representing the natural population analysis of species **II**.

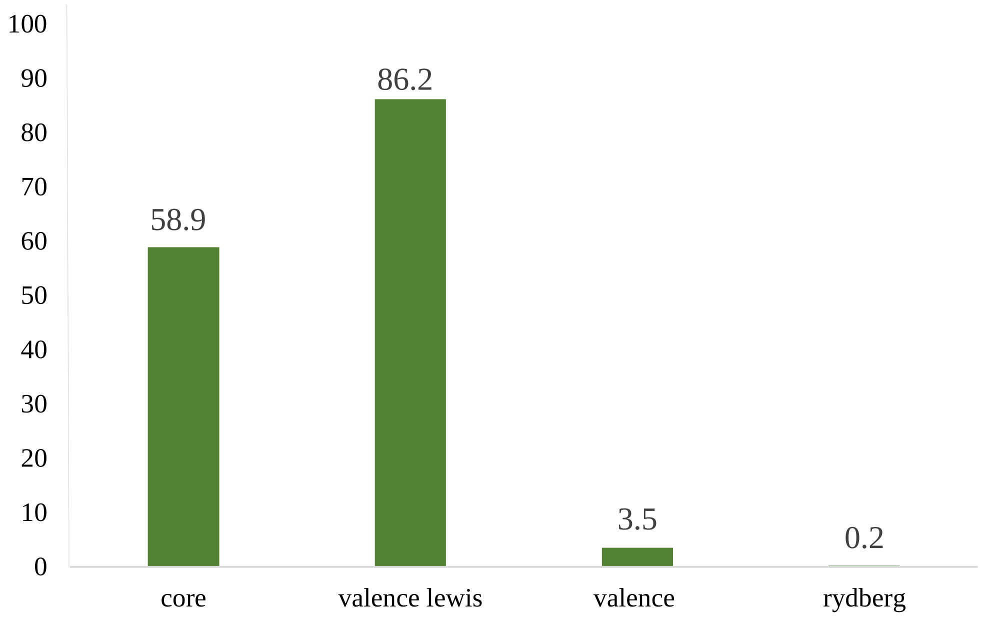

Fig. S14. Bar diagram representing the natural population analysis of species **III**.

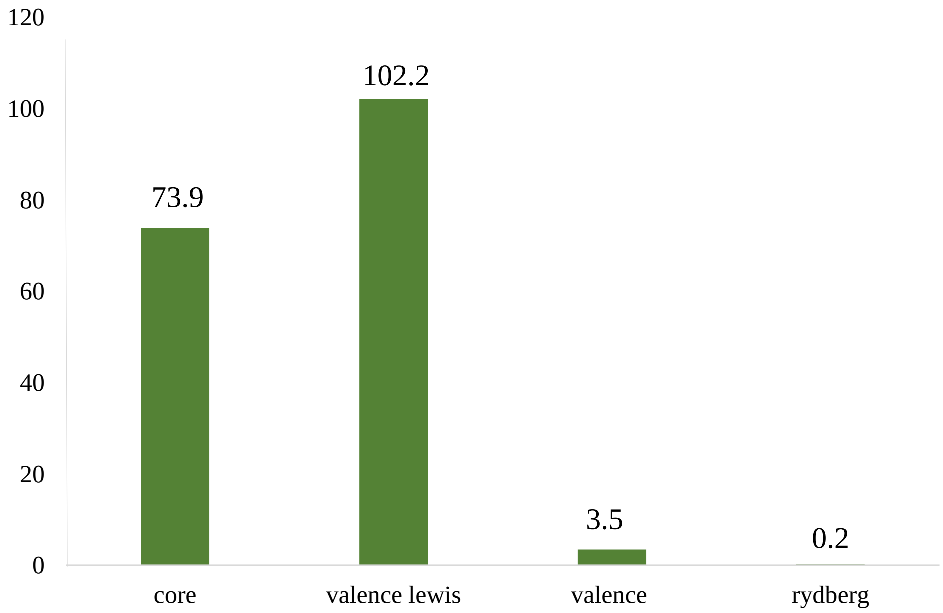

Fig. S15. Bar diagram representing the natural population analysis of species ^{3}**IV**.

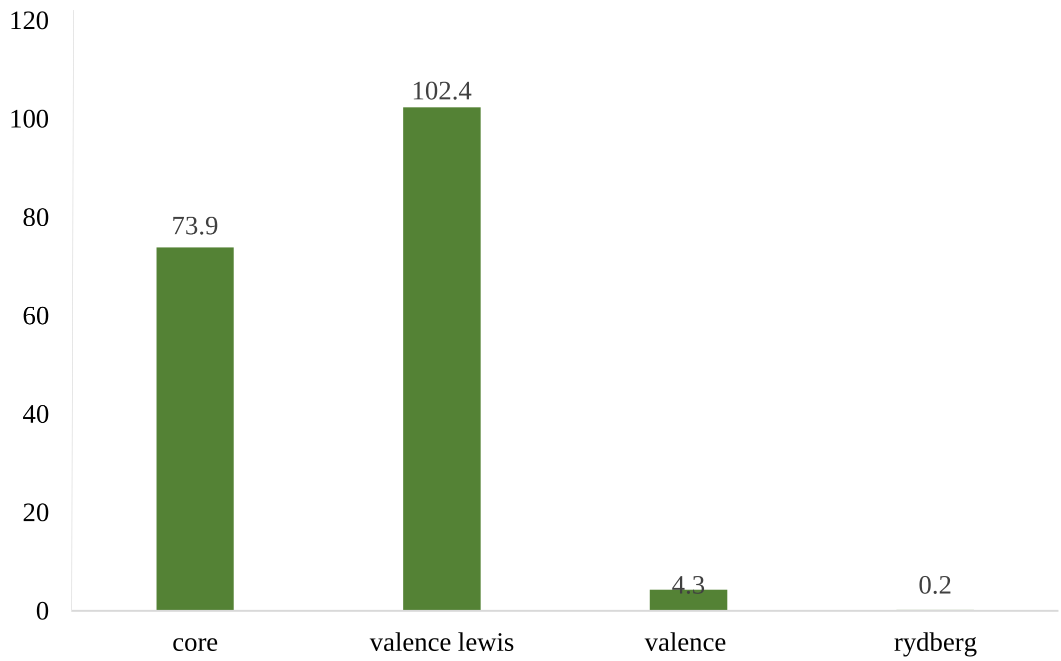

Fig. S16. Bar diagram representing the natural population analysis of species ^{1}**IV**.

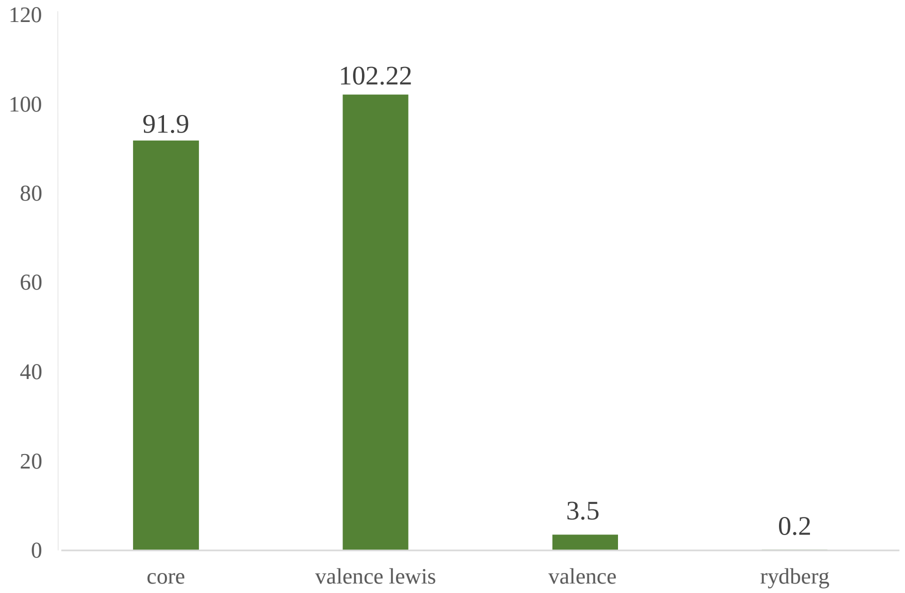

Fig. S17. Bar diagram representing the natural population analysis of species ^{3}**V**.

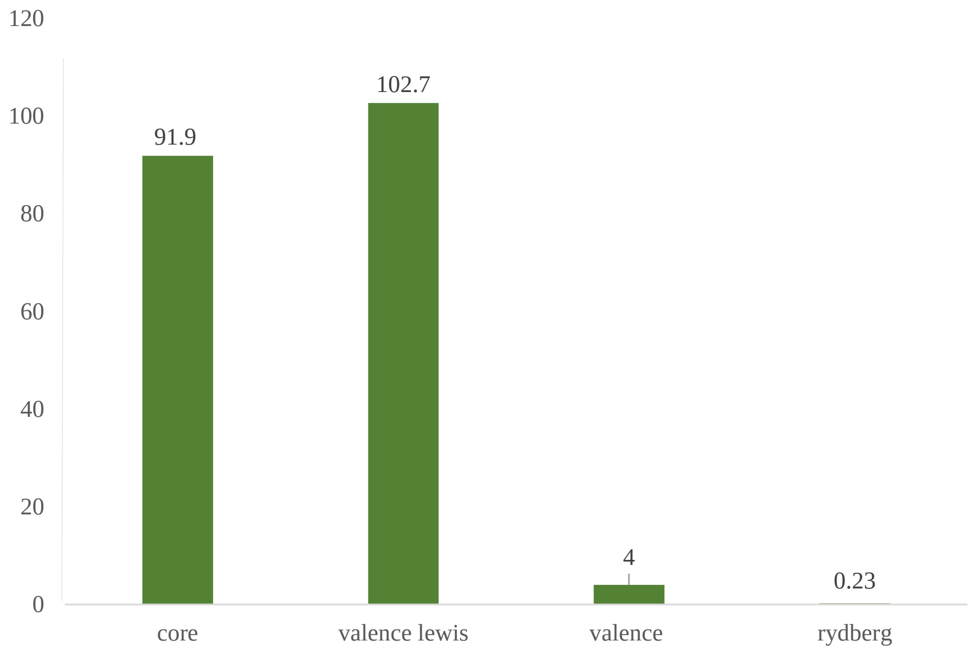

Fig. S18. Bar diagram representing the natural population analysis of species ^{1}**V**.

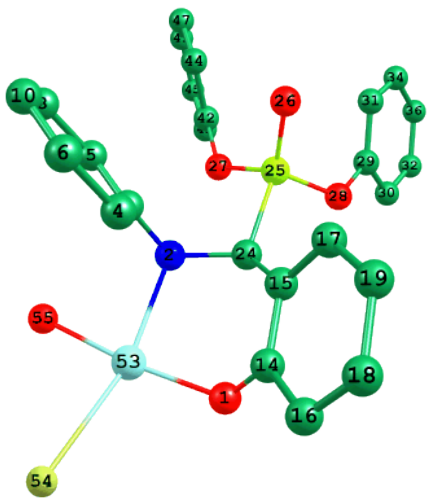

Fig. S19. Visualization of species **I** with atom numbering for study of second order perturbation theory analysis of Fock matrix in NBO basis.

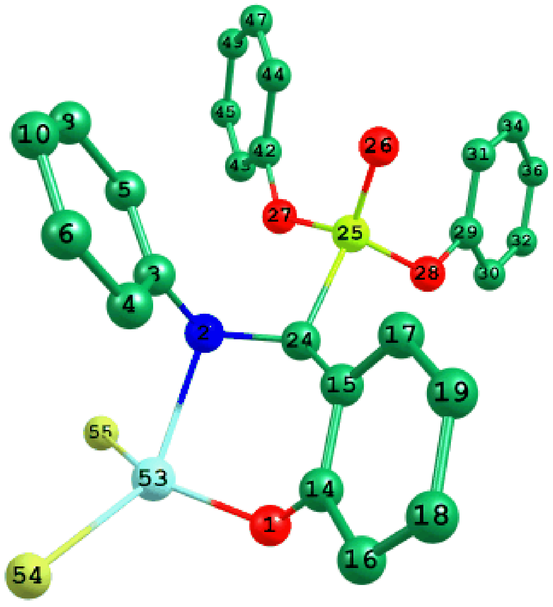

Fig. S20. Visualization of species **II** with atom numbering for study of second order perturbation theory analysis of Fock matrix in NBO basis.

Table S2. Perturbation theory energy analysis of species **II**.

Donar NBO (i)	Acceptor NBO (i)	$E^{(2a)}$kcal/mol	$E(j)-E(i)^{b}$(a.u.)	$F(i, j)^{c}$(a.u.)
π (C3 - C4)	π* (C5 - C8)	10.55	0.27	0.068
π (C3 - C4)	π* (C6 - C10)	9.98	0.27	0.066
π (C5 - C8)	π* (C3 - C4)	9.81	0.28	0.067
π (C5 - C8)	π* (C6 - C10)	9.79	0.27	0.066
π (C6 - C10)	π* (C3 - C4)	9.95	0.28	0.067
π (C6 - C10)	π* (C5 - C8)	10.23	0.27	0.045
π (C29 - C30)	π* (C31 - C34)	9.25	0.28	0.064
π (C29 - C30)	π* (C32 - C36)	10.97	0.27	0.069
π (C31 - C34)	π* (C29 - C30)	11.05	0.27	0.070
π (C31 - C34)	π* (C32 - C36)	9.34	0.27	0.064
π (C32 - C36)	π* (C29 - C30)	9.01	0.27	0.063
π (C32 - C36)	π* (C31 - C34)	10.49	0.27	0.068
π (C42 - C43)	π* (C44 - C47)	9.40	0.28	0.065
π (C42 - C43)	π* (C45 - C49)	10.76	0.28	0.069
π (C45 - C49)	π* (C42 - C43)	9.12	0.27	0.063
π (C45 - C49)	π* (C44 - C47)	10.49	0.27	0.068
n (O1)	n*(Cu 53)	13.02	0.75	0.125
n (O26)	σ* (C24 - P25)	7.02	0.38	0.065
n (O26)	σ* (P25 - O27)	8.03	0.37	0.069
n (O26)	σ* (P25 - O28)	8.72	0.36	0.071
n (O27)	π* (C42 - C43)	8.60	0.36	0.075

n (O28)	π* (C29 - C30)	9.11	0.35	0.076
n(Br 55)	n*(Cu 53)	5.95	0.99	0.103
n(Br 55)	n*(Cu 53	8.28	0.55	0.088
n(Br 55)	n*(Cu53)	43.12	0.51	0.193

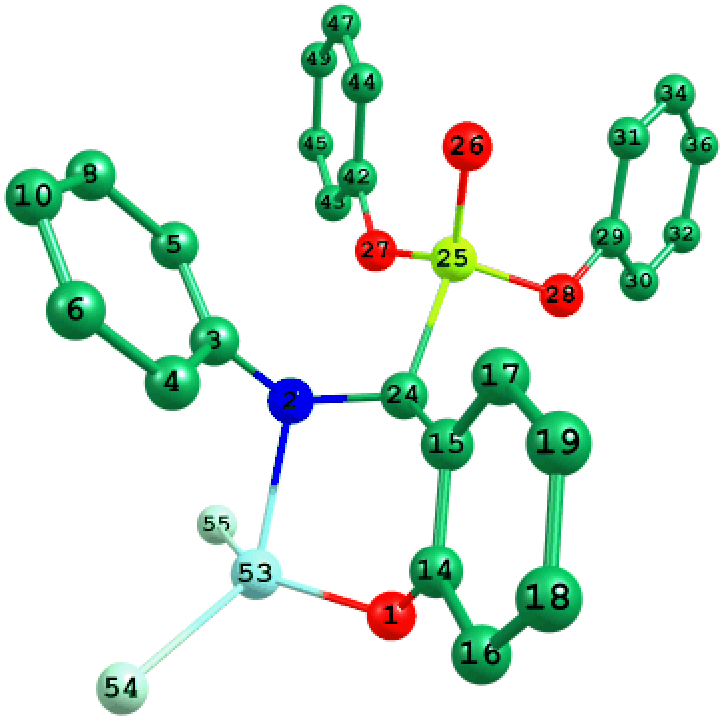

Fig. S21. Visualization of species **III** with atom numbering for study of second order perturbation theory analysis of Fock matrix in NBO basis.

Table S3. Perturbation theory energy analysis of species **III**.

**Donar NBO (i)**	**Acceptor NBO (i)**	**$E^{(2a)}$kcal/mol**	**E(j)-E(i)^{b}(a.u.)**	**F(i, j)^{c}(a.u.)**
σ (O1 -Cu 53)	σ*(Cu 53 -Cl 54)	4.09	0.44	0.054
π (C3 - C4)	π* (C5 - C8)	10.61	0.27	0.068
π (C3 - C4)	π* (C6 - C10)	10.00	0.27	0.066
π (C5 - C8)	π* (C3 - C4)	9.78	0.28	0.067
π (C5 - C8)	π* (C6 - C10)	9.80	0.27	0.066
π (C6 - C10)	π* (C3 - C4)	9.93	0.28	0.067
π (C6 - C10)	π* (C5 - C8)	10.22	0.27	0.066
π (C29 - C30)	π* (C31 - C34)	9.30	0.28	0.064
π (C29 - C30)	π* (C32 - C36)	10.87	0.27	0.069
π (C31 - C34)	π* (C29 - C30)	10.96	0.27	0.069
π (C31 - C34)	π* (C32 - C36)	9.43	0.27	0.064
π (C32 - C36)	π* (C29 - C30)	9.05	0.27	0.063
π (C32 - C36)	π* (C31 - C34)	10.41	0.27	0.068
π (C42 - C43)	π* (C44 - C47)	9.37	0.28	0.065
π (C42 - C43)	π* (C45 - C49)	10.79	0.28	0.069
π (C44 - C47)	π* (C42 - C43)	11.00	0.27	0.070
π (C44 - C47)	π* (C45 - C49)	9.39	0.27	0.064
π (C45 - C49)	π* (C42 - C43)	9.09	0.27	0.063
π (C45 - C49)	π* (C44 - C47)	10.49	0.27	0.068
n (O1)	n* (Cu 53)	12.94	0.76	0.125
n (N2)	σ* (C24 – P 25)	7.17	0.41	0.070
n (C19)	π* (C15 - C17)	32.59	0.14	0.103
n (C19)	π* (C16 - C18)	30.07	0.14	0.100
n (O 26)	σ* (P 25 - O 28)	9.96	0.36	0.076
n (O 27)	π* (C42 - C43)	9.00	0.36	0.077
n (O 28)	π* (C29 - C30)	7.79	0.36	0.072
n (Cl55)	n* (Cu53)	7.63	0.55	0.085
n (Cl55)	n* (Cu53)	42.16	0.55	0.196

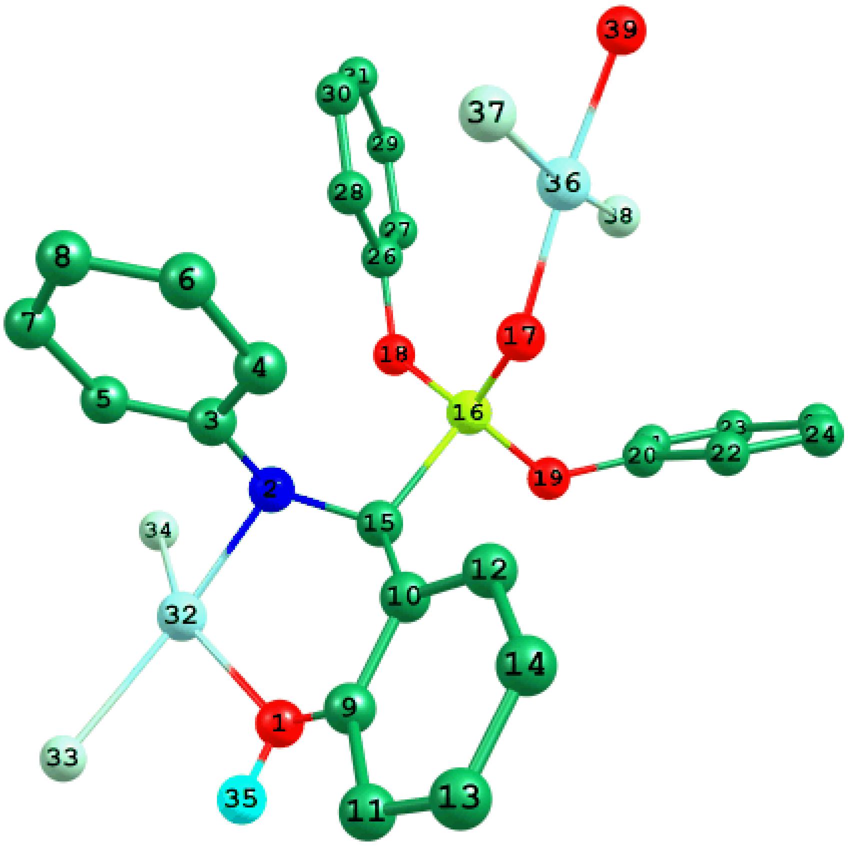

Fig. S22. Visualization of species **IV** with atom numbering for study of second order perturbation theory analysis of Fock matrix in NBO basis.

Table S4. Perturbation theory energy analysis of species ^{3}**IV**.

Donar NBO (i)	Acceptor NBO (i)	E$^{(2a)}$kcal/mol	E(j)-E(i)^{b}(a.u.)	F(i, j)^{c}(a.u.)
π (C3-C4)	π *(C5-C8)	8.74	0.29	0.064
π (C3-C4)	π *(C6-C10)	8.95	0.30	0.065
π (C5-C8)	π *(C3-C4)	10.59	0.26	0.068
π (C5-C8)	π *(C6-C10)	9.27	0.28	0.065
π (C6-C10)	π *(C3-C4)	10.53	0.26	0.066
π (C6-C10)	π *(C5-C8)	10.01	0.27	0.067
π (C14-C15)	π *(C16-C18)	8.24	0.29	0.062
π (C14-C15)	π *(C17-C19)	10.43	0.30	0.071
π (C16-C18)	π *(C14-C15)	12.22	0.26	0.073
π (C16-C18)	π *(C17-C19)	8.74	0.28	0.063
π (C17-C19)	π *(C14-C15)	9.59	0.26	0.064
π (C17-C19)	π *(C16-C18)	11.29	0.27	0.070
π (C29-C30)	π *(C31-C34)	9.53	0.29	0.066

π (C29-C30)	π *(C32-C36)	9.49	0.29	0.066
π (C31-C34)	π *(C29-C30)	11.17	0.27	0.069
π (C31-C34)	π *(C32-C36)	9.57	0.27	0.065
π (C32-C36)	π *(C29-C30)	10.05	0.26	0.065
π (C32-C36)	π *(C31-C34)	10.43	0.27	0.067
π (C42-C43)	π *(C44-C47)	9.96	0.28	0.067
π (C42-C43)	π *(C45-C49)	9.60	0.29	0.066
π (C44-C47)	π *(C42-C43)	11.13	0.27	0.069
π (C44-C47)	π *(C45-C49)	9.39	0.28	0.064
π (C45-C49)	π *(C42-C43)	10.07	0.26	0.065
π (C45-C49)	π *(C44-C47)	10.71	0.27	0.068
n (O 1)	n*(Cu 53)	9.95	0.75	0.110
n (N2)	n*(Cu 53)	14.76	0.71	0.136

Table S5. Perturbation theory energy analysis of species ^{1}**IV**.

**Donar NBO (i)**	**Acceptor NBO (i)**	**E$^{(2a)}$kcal/mol**	**E(j)-E(i)^{b}(a.u.)**	**F(i, j)^{c}(a.u.)**
π (C3 - C4)	π*(C5 - C8)	9.05	0.29	0.065
π (C3 - C4)	π*(C6 - C10)	8.86	0.30	0.065
σ (C4 - C6)	σ *(n2 - C3)	3.11	1.00	0.071
π (C5 - C8)	π*(C3 - C4)	10.46	0.26	0.067
π (C5 - C8)	π*(C6 - C10)	9.36	0.28	0.065
π (C6 - C10)	π*(C3 - C4)	10.65	0.26	0.067
π (C6 - C10)	π*(C5 - C8)	10.04	0.27	0.067
π (C14 - C15)	π*(C16 - C18)	8.37	0.29	0.062
π (C14 - C15)	π*(C17 - C19)	10.29	0.30	0.070
π (C16 - C18)	π*(C14 - C15)	12.10	0.26	0.073
π (C16 - C18)	π*(C17 - C19)	8.80	0.28	0.063
π (C17 - C19)	π*(C14 - C15)	9.74	0.26	0.064
π (C17 - C19)	π*(C16 - C18)	11.23	0.27	0.069
π (C29 - C30)	π*(C31 - C34)	9.53	0.29	0.066
π (C29 - C30)	π*(C32 - C36)	9.48	0.29	0.066
π (C31 - C34)	π*(C29 - C30)	11.16	0.27	0.069
π (C31 - C34)	π*(C32 - C36)	9.55	0.28	0.065
π (C42 - C43)	π*(C44 - C47)	9.98	0.28	0.067
π (C42 - C43)	π*(C45 - C49)	9.59	0.29	0.066
π (C44 - C47)	π*(C42 - C43)	11.11	0.27	0.069
π (C44 - C47)	π*(C45 - C49)	9.35	0.28	0.064
π (C45 - C49)	π*(C42 - C43)	10.06	0.26	0.065
π (C45 - C49)	π*(C44 - C47)	10.77	0.27	0.068
n (O1)	π*(C14 - C15)	3.08	0.52	0.055
n (O1)	π*(C14 - C15)	4.54	0.49	0.064

n (N2)	π*(C3 - C4)	5.57	0.39	0.059
n (N 2)	σ *(C24 - P 25)	4.77	0.45	0.062
n (O 26)	σ * (P 25 - O 28)	8.92	0.41	0.078
n (O 28)	σ * (P 25 - O 27)	5.31	0.46	0.063
n (O1)	n*(6) Cu 53	7.00	0.79	0.095
n (O1)	n*(6) Cu 53	20.55	0.77	0.161
n (N2)	n*(5) Cu 53	19.15	0.68	0.151
n (N2)	n*(Cu 53)	5.35	0.64	0.078
n (O26)	n*(Cu 57)	6.45	0.69	0.088
n (O26)	n*(Cu 57)	11.38	1.16	0.146
n (O26)	n*(Cu 57)	26.24	0.45	0.138
n (O26)	n*(Cu 57)	16.80	0.92	0.163
n (Cl 54)	n*(Cu 53)	2.99	1.01	0.072
n (Cl 54)	n*(Cu 53)	5.77	0.77	0.087
n (Cl 55)	n*(Cu 53)	3.62	0.79	0.070
σ (Cu 57 -Cl 58)	n*(Cu 57)	2.05	0.34	0.035
σ (Cu 57 -Cl 58)	σ *(Cu 57 -Cl 59)	6.21	0.48	0.070
σ (Cu 57 -Cl 59)	n*(Cu 57)	2.16	0.34	0.036
σ(Cu 57 -Cl 59)	σ *(Cu 57 -Cl 58)	6.81	0.49	0.074
n (O 60)	n*(Cu 57)	26.13	0.65	0.168
n (O 60)	n*(Cu 57)	20.58	1.12	0.195

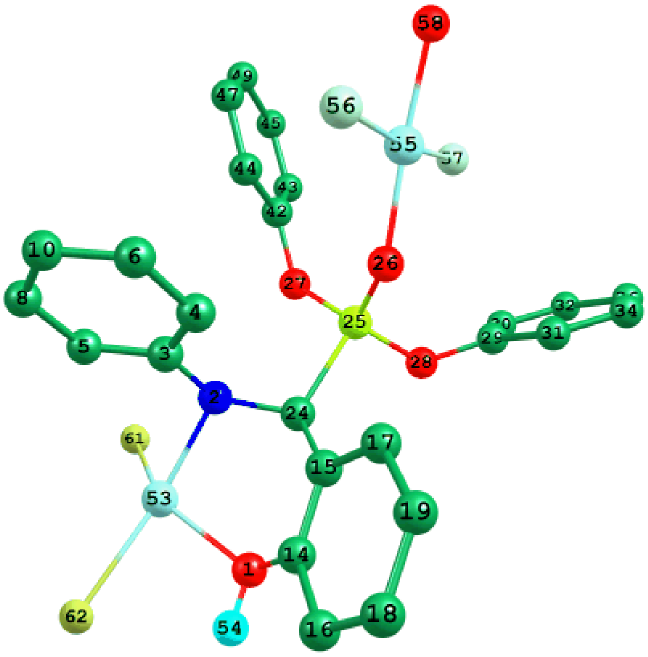

Fig. S23. Visualization of species **V** with atom numbering for study of second order perturbation theory analysis of Fock matrix in NBO basis.

Table S6. Perturbation theory energy analysis of species ^{3}**V**.

Donar NBO (i)	Acceptor NBO (i)	$E^{(2a)}$kcal/mol	$E(j)-E(i)^{b}$(a.u.)	$F(i, j)^{c}$(a.u.)
π (C3-C4)	π* (C5-C8)	8.75	0.29	0.064
π (C3-C4)	π* (C6-C10)	8.99	0.30	0.066
π (C5-C8)	π* (C3-C4)	10.59	0.27	0.068
π (C5-C8)	π* (C6-C10)	9.18	0.28	0.064
π (C6-C10)	π* (C3-C4)	10.44	0.26	0.066
π (C6-C10)	π* (C5-C8)	10.11	0.27	0.067
π (C14-C15)	π* (C16-C18)	8.25	0.29	0.062
π (C14-C15)	π* (C17-C19)	10.49	0.30	0.071
π (C16-C18)	π* (C14-C15)	12.22	0.26	0.073
π (C16-C18)	π* (C17-C19)	8.73	0.28	0.063
π (C17-C19)	π* (C14-C15)	9.57	0.26	0.064
π C17-C19)	π* (C16-C18)	11.32	0.27	0.070
π (C29-C30)	π* (C31-C34)	9.55	0.28	0.066
π (C29-C30)	π* (C32-C36)	9.52	0.29	0.066
π (C31-C34)	π* (C29-C30)	11.14	0.27	0.069
π (C31-C34)	π* (C32-C36)	9.55	0.28	0.065
π (C32-C36)	π* (C29-C30)	10.04	0.26	0.065
π (C32-C36)	π* (C31-C34)	10.46	0.27	0.067
π (C42-C43)	π* (C44-C47)	9.93	0.28	0.067
π (C42-C43)	π* (C45-C49)	9.59	0.29	0.066
π (C44-C47)	π* (C42-C43)	11.11	0.27	0.069
π (C44-C47)	π* (C45-C49)	9.38	0.28	0.064
π (C45-C49)	π* (C42-C43)	10.08	0.26	0.065
π (C45-C49)	π* (C44-C47)	10.73	0.27	0.068
n (O1)	π* (C14-C15)	6.35	0.44	0.072
n (O26)	σ* P 25-O 28	8.99	0.42	0.078
π* (C3-C4)	π* (C5-C8)	125.12	0.01	0.077
π* (C3-C4)	π* (C6-C10)	89.62	0.01	0.078
π* (C14-C15)	π* (C16-C18)	134.02	0.01	0.079
π* (C14-C15)	π* (C17-C19)	74.41	0.02	0.077
n (O 1)	n*(Cu 53)	7.36	0.69	0.092
n (O 1)	n*(Cu 53)	7.21	0.75	0.093
n (N2)	n*(Cu 53)	14.75	0.68	0.132
n (O 26)	n*(Cu 55)	12.97	1.22	0.160
n (O 26	n*(Cu 55)	14.02	0.49	0.105
n (O 26)	n*(Cu 55)	14.15	0.93	0.149
σ (Cu 55 -Br 62)	n*(Cu 53)	17.78	0.52	0.124
n (Br 62)	n*(Cu 53)	14.25	0.65	0.126
n (Cl 56)	n*(Cu 55)	21.07	0.33	0.108
n (Cl 56)	n*(Cu 55)	25.02	0.61	0.163
n (Cl 56)	σ *(Cu 55 -Br 62)	32.47	0.02	0.038

n (Cl 57)	n*(Cu 55)	24.43	0.33	0.117
n (Cl 57)	n*(Cu 55)	22.92	0.61	0.157
n (Cl 57)	σ* (Cu 55 -Br 62)	39.83	0.01	0.039
n (O 58)	n*(Cu 55)	17.94	0.69	0.146
n (O 58)	n*(Cu 55)	19.70	1.14	0.190

Table S7. Perturbation theory energy analysis of species ^{1}**V**.

**Donar NBO (i)**	**Acceptor NBO (i)**	**$E^{(2a)}$kcal/mol**	**E(j)-E(i)^{b}(a.u.)**	**F(i, j)^{c}(a.u.)**
π (C3-C4)	π* (C5-C8)	8.75	0.29	0.064
π (C3-C4)	π* (C6-C10)	8.99	0.30	0.066
π (C5-C8)	π* (C3-C4)	10.59	0.27	0.068
π (C5-C8)	π* (C6-C10)	9.18	0.28	0.064
π (C6-C10)	π* (C3-C4)	10.44	0.26	0.066
π (C6-C10)	π* (C5-C8)	10.11	0.27	0.067
π (C14-C15)	π* (C16-C18)	8.25	0.29	0.062
π (C14-C15)	π* (C17-C19	10.49	0.30	0.071
π (C16-C18)	π* (C14-C15)	12.22	0.26	0.073
π (C16-C18)	π* (C17-C19)	8.73	0.28	0.063
π (C17-C19)	π* (C14-C15)	9.57	0.26	0.064
π (C17-C19)	π* (C16-C18)	11.32	0.27	0.070
π (C29-C30)	π* (C31-C34)	9.55	0.28	0.066
π (C29-C30)	π* (C32-C36)	9.52	0.29	0.066
π (C31-C34)	π* (C29-C30)	11.14	0.27	0.069
π (C31-C34)	π* (C32-C36)	9.55	0.28	0.065
π (C32-C36)	π* (C29-C30)	10.04	0.26	0.065
π (C32-C36)	π* (C31-C34)	10.46	0.27	0.067
π (C42-C43)	π* (C44-C47)	9.93	0.28	0.067
π (C42-C43)	π* (C45-C49)	9.59	0.29	0.066
π (C44-C47)	π* (C42-C43)	11.11	0.27	0.069
π (C44-C47)	π* (C45-C49)	9.38	0.28	0.064
π (C45-C49)	π* (C42-C43)	10.08	0.26	0.065
π (C45-C49)	π* (C44-C47)	10.73	0.27	0.068
n (O 26)	σ (P 25-O 28)	8.99	0.42	0.078
n (O 1)	n*(Cu 53)	7.36	0.69	0.092
n (O 1)	n*(Cu 53)	7.21	0.75	0.093
n (N 2)	n*(Cu 53)	14.75	0.68	0.132

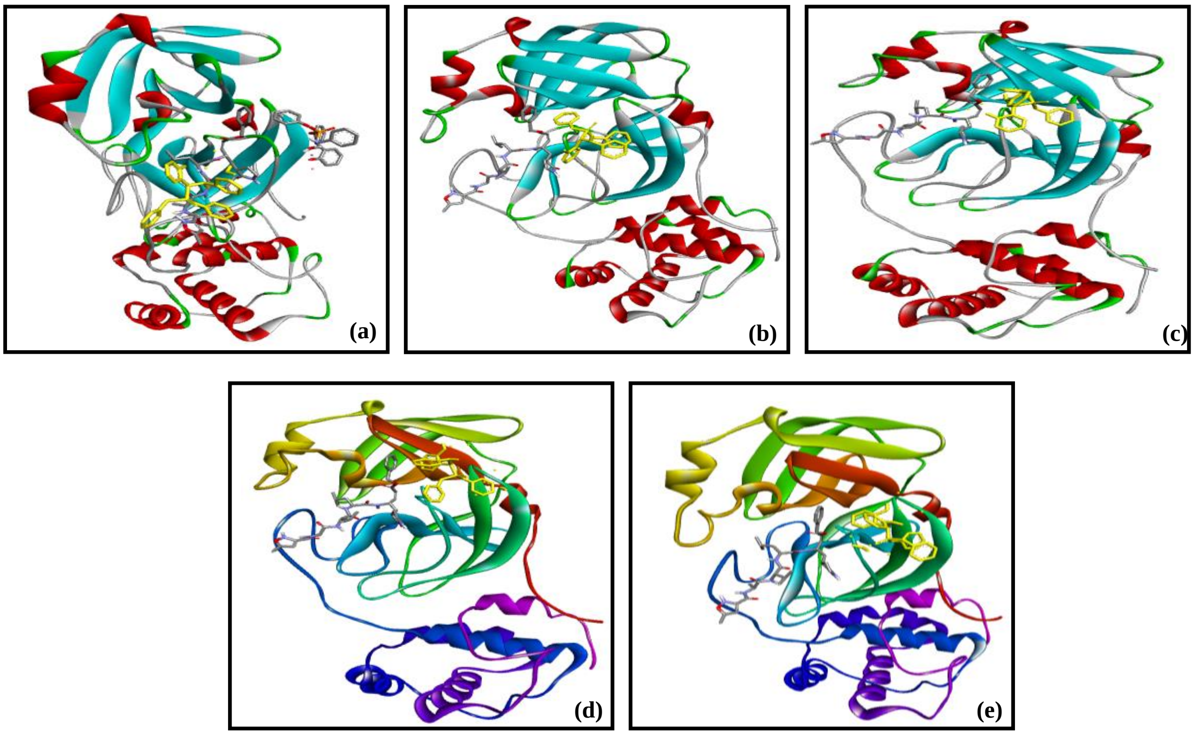

Fig. S24. Orientation of species in the active sites of the SARS-CoV-2 Protease (6LU7): a) Species **I**, (b) Species **II**, (c) Species **III**, (d) Species ^{3}**IV** and (e) Species ^{3}**V**.

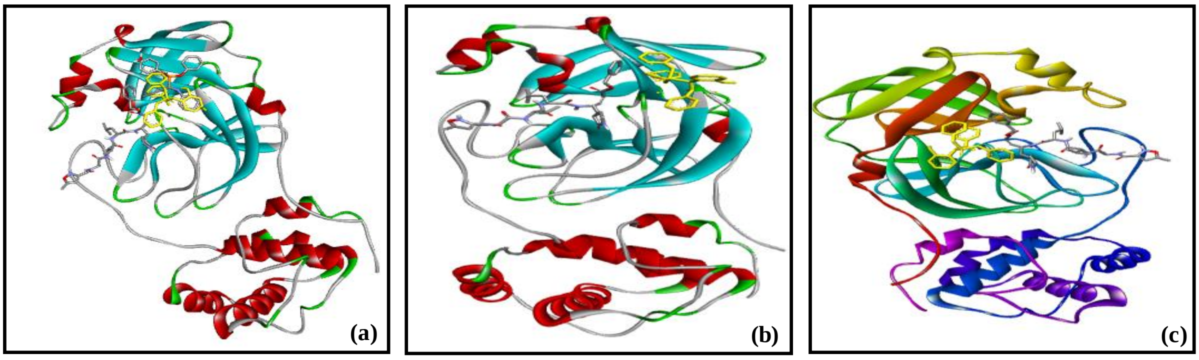

Fig. S25. Orientation of species in the active sites of the SARS-CoV-2 Protease (6LU7): (a) Species ^{3}**IV**, (b) Species ^{3}**V** and (c) Ligand.

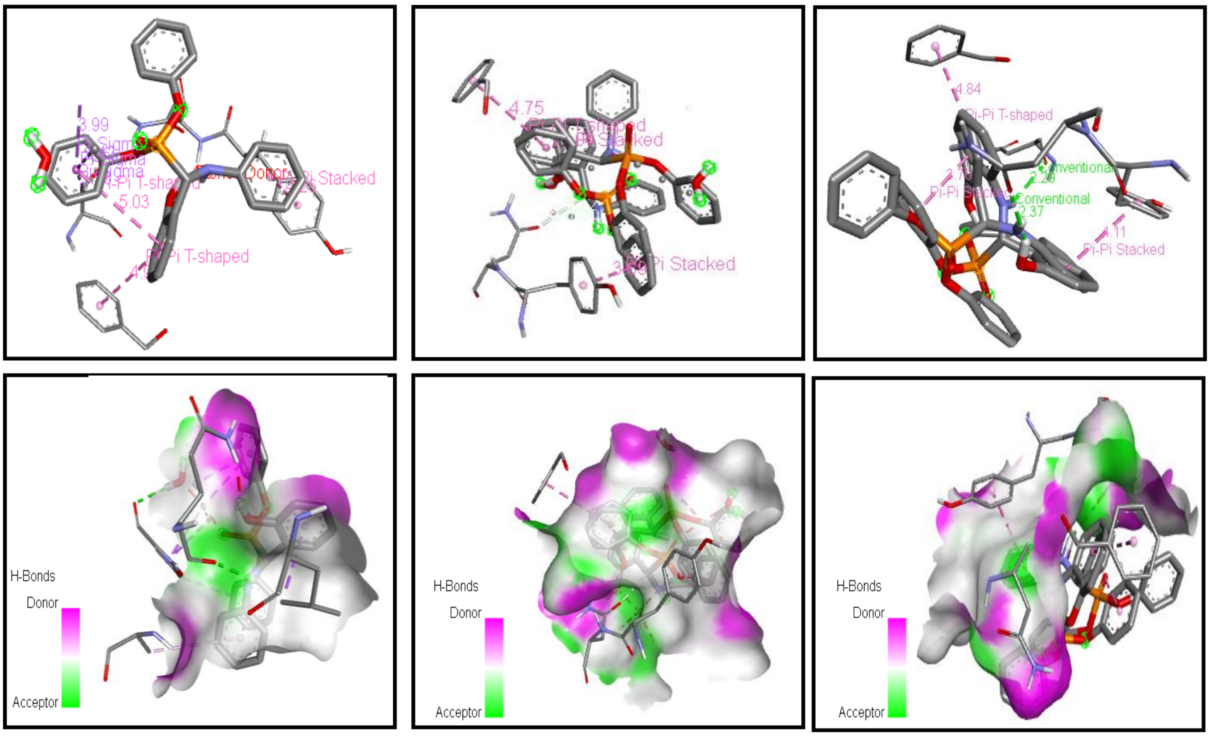

(a) (b) (c)

Fig. S26. Details of interactions of species SARS-CoV-2 Protease (6LU7): with protein (6LU7): (a) Species ^{3}**IV**, (b) Species ^{3}**V** and (c) Ligand.

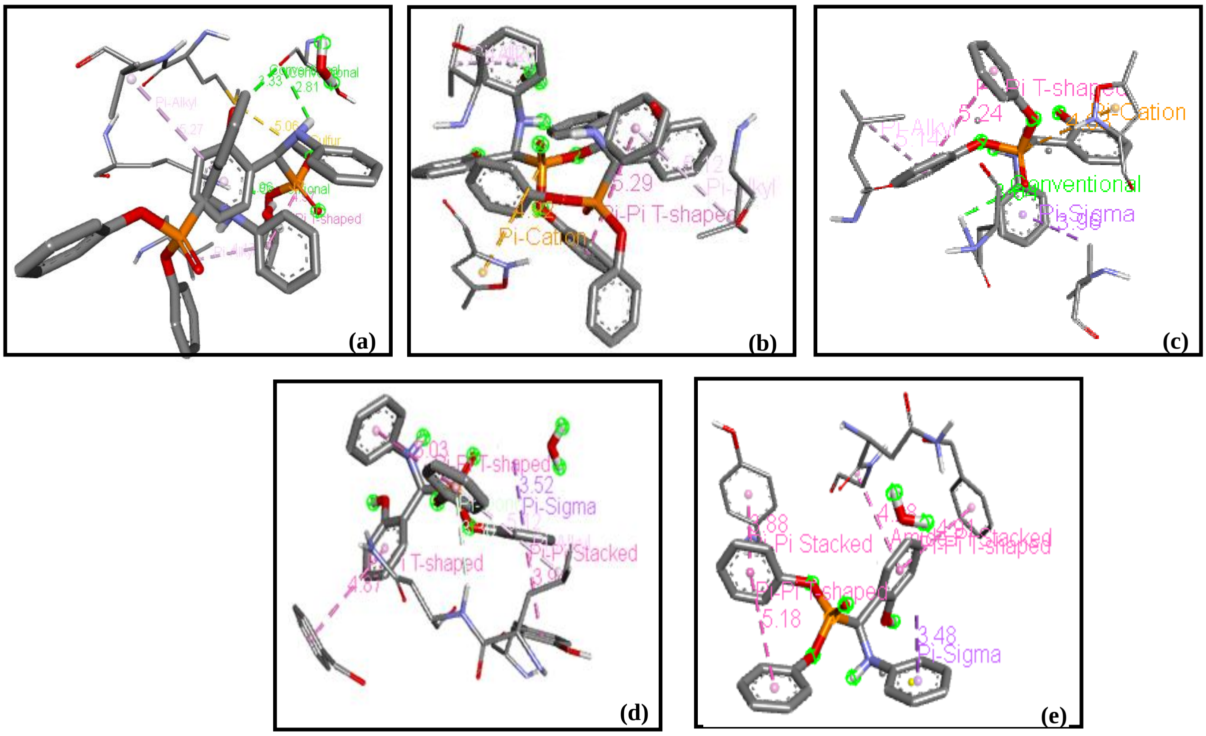

Fig. S27. Details of interactions of ligand with SARS-CoV-2 Protease (6LU7).

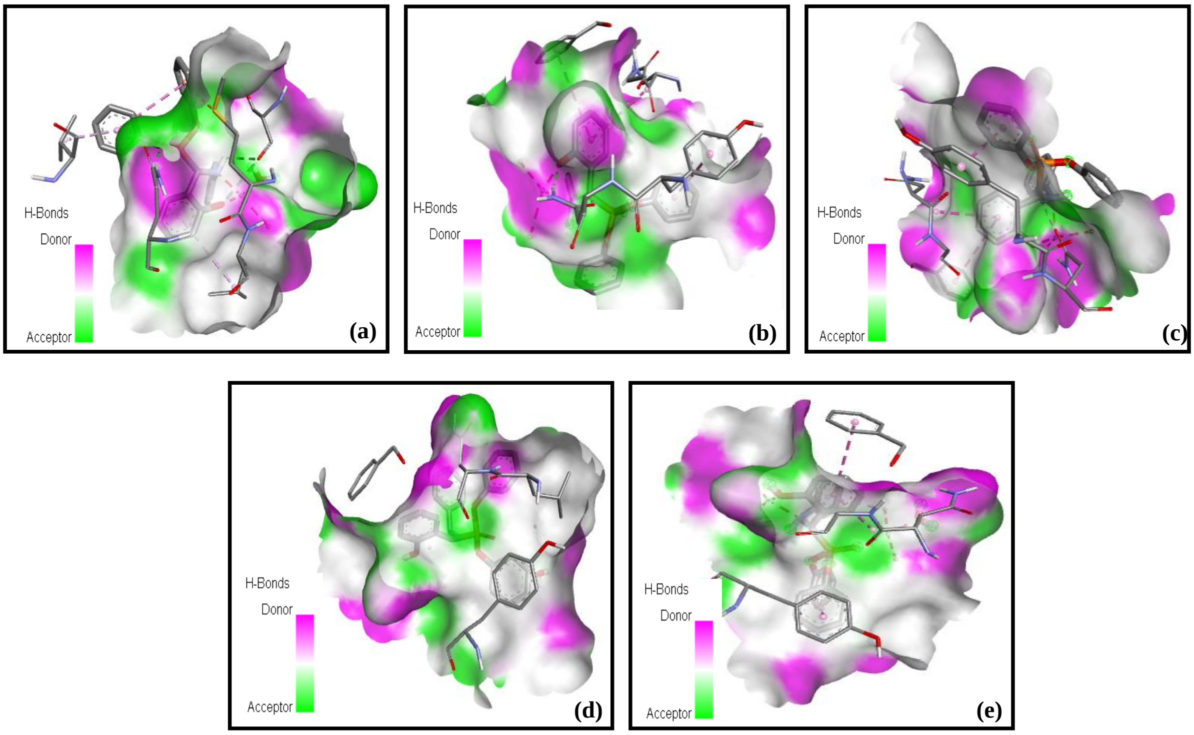

Fig. S28. Different interactions of docked pose of ligand with the SARS-CoV-2 Protease (6LU7).

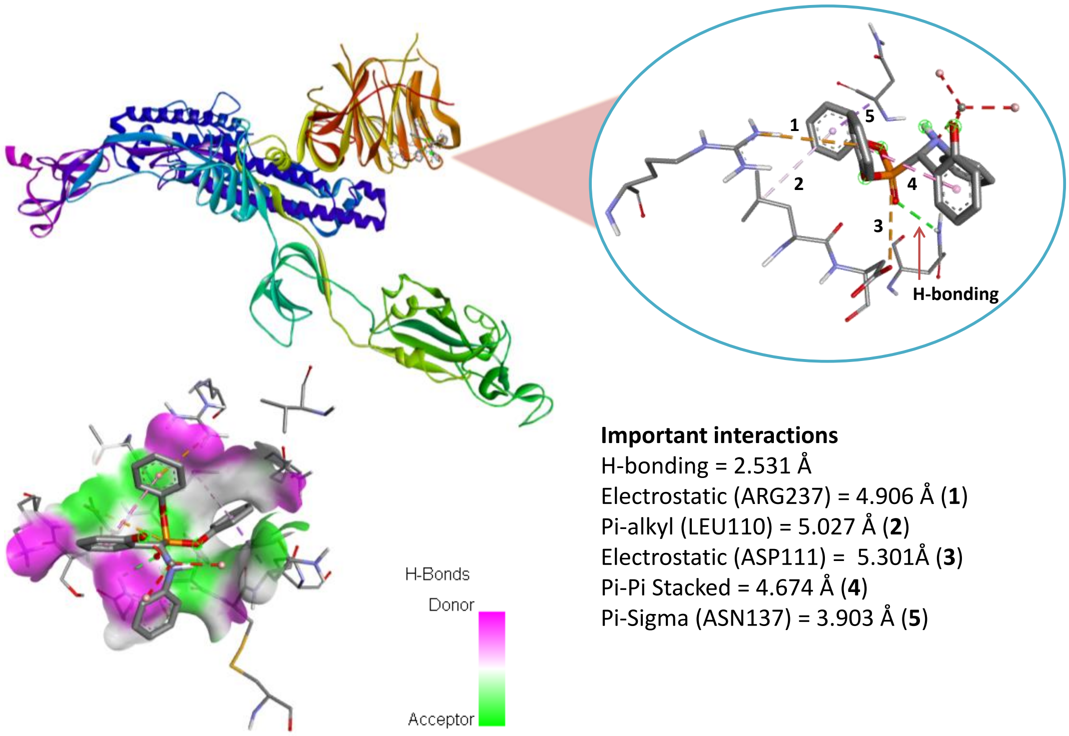

Fig.S29. Molecular docking study of species **II** with the SARS-CoV-2 Omicron variant protease (7T9K) (FBE = -5.4 kcal/mol).

Different interactions such as H-bonding, electrostatic and non-covalent interactions are present in favourable docked pose of ligand-receptor. Docked pose bear interactions with

amino-acid residues such as arginine, leucine, aspartic acid and asparagine. Among mononuclear species, the bond length for H-bonding is highest in case of species III.

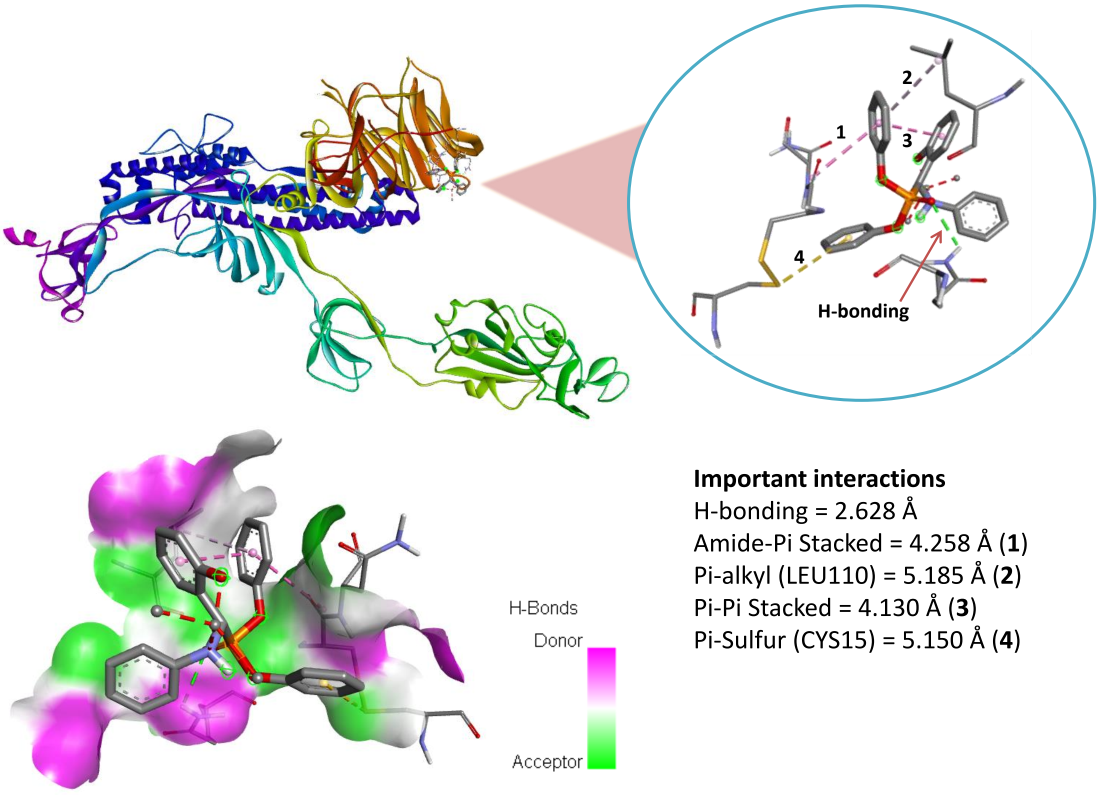

Fig. S30. Molecular docking study of species **III** with the SARS-CoV-2 Omicron variant protease (7T9K) (FBE = -5.4 kcal/mol).

Favourable docked pose showed interactions such as H-bonding and non-covalent interactions with amino acid residues such as leucine and cysteine.

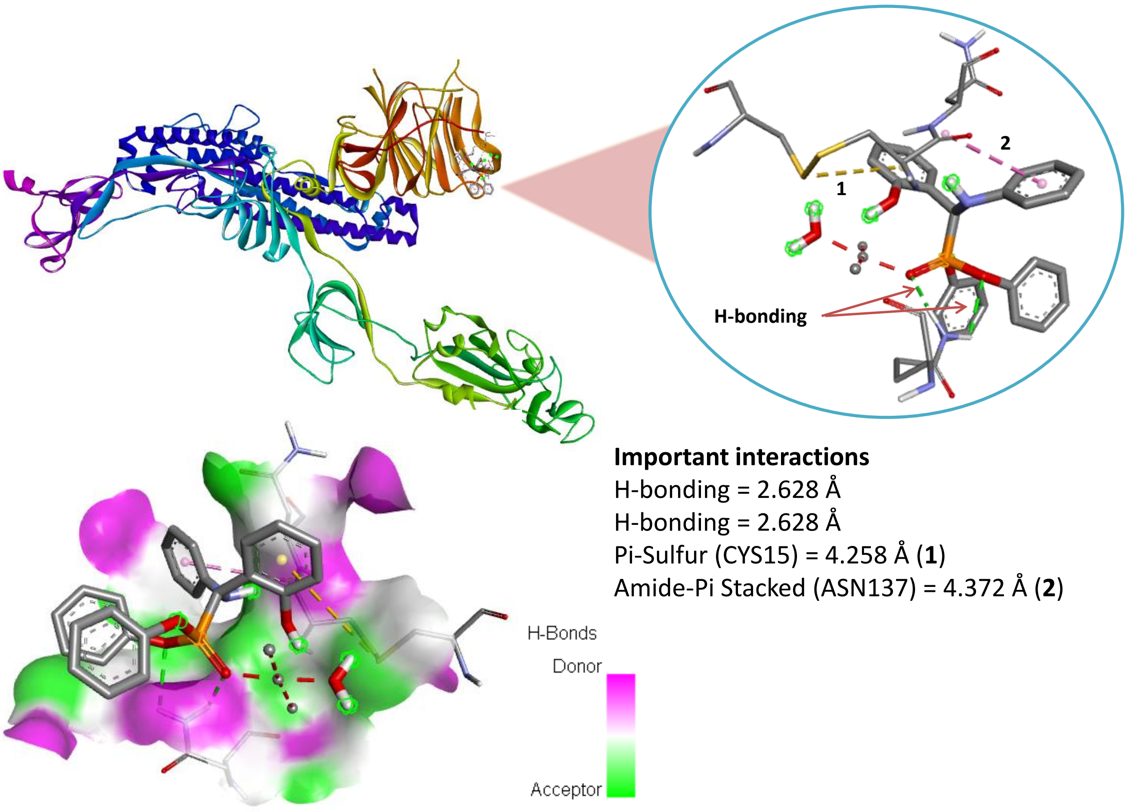

Fig. S31. Molecular docking study of species ^{3}**IV** with the SARS-CoV-2 Omicron variant protease (7T9K) (FBE = -4.9 kcal/mol).

Favourable docked pose showed interactions such as H-bonding (two H- bonds) and non-covalent interactions with amino acid residues such as cysteine and asparagine.

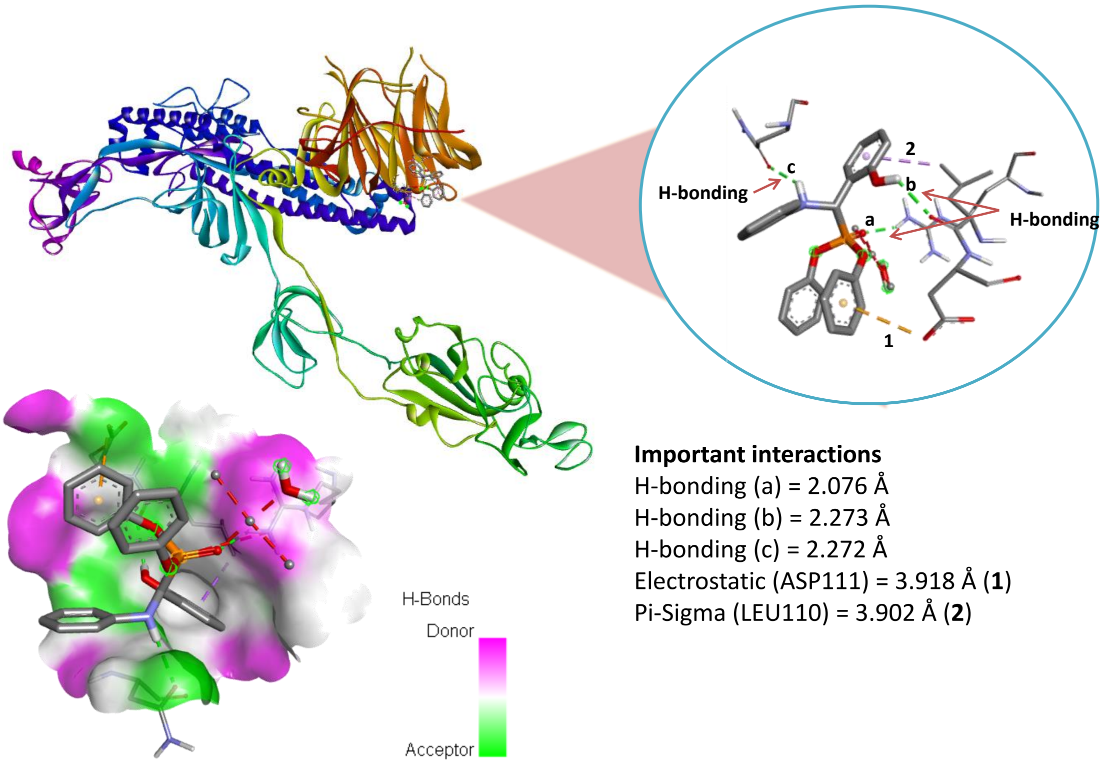

Fig. S32. Molecular docking study of species [1]**IV** with the SARS-CoV-2 Omicron variant protease (FBE = -5.2 kcal/mol).

Favourable docked pose showed interactions such as H-bonding (two H- bonds), electrostatic and non-covalent interaction with amino acid residues such as asparagine and leucine.

.

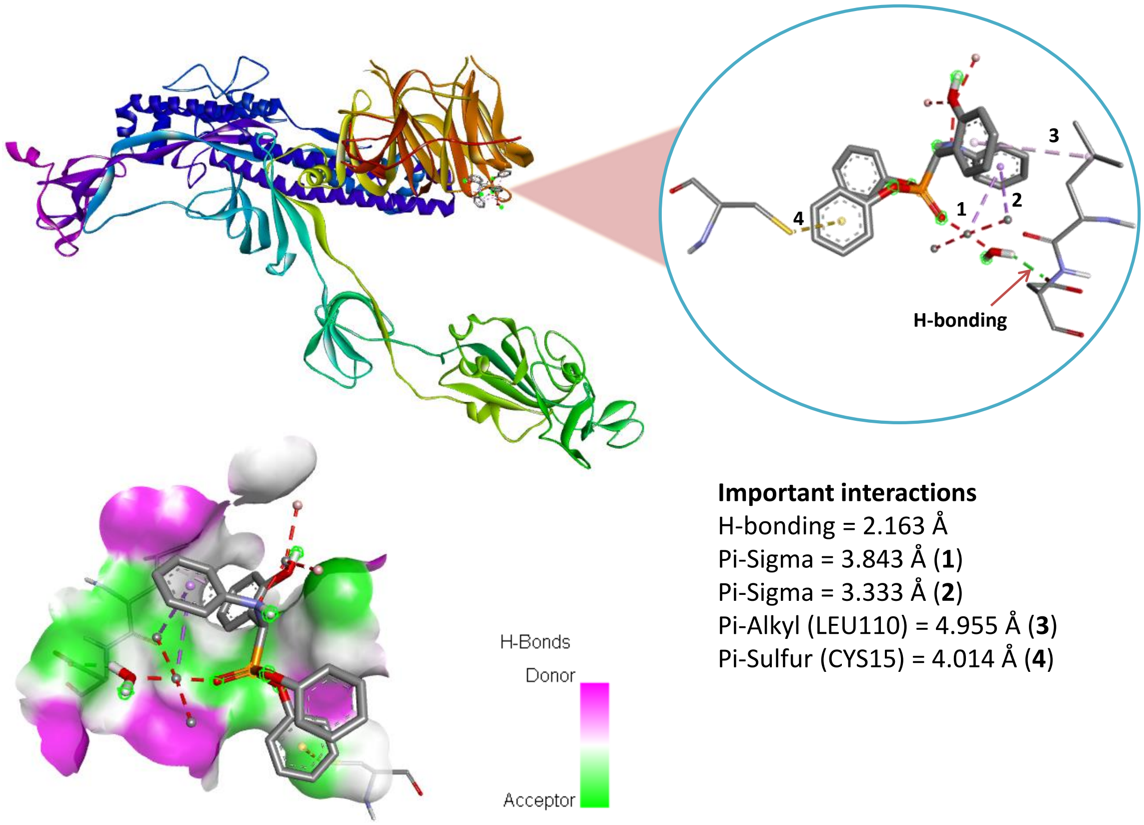

Fig. S33. Molecular docking studies of species ^{3}**V** with the SARS-CoV-2 Omicron variant protease (7T9K) (FBE = -5.3 kcal/mol).

Favourable docked pose showed interactions such as H-bonding and non-covalent interaction with amino acid residues such as leucine and cysteine.

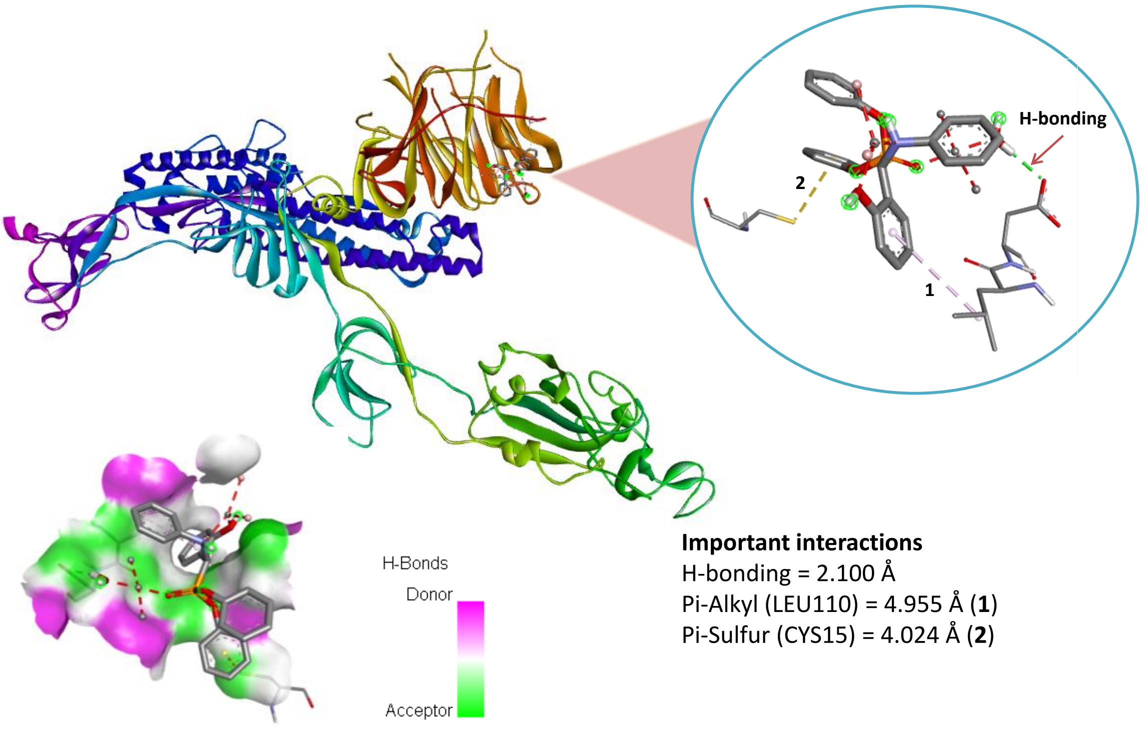

Fig. S34. Molecular docking studies species ^{1}**V** with the SARS-CoV-2 Omicron variant protease (7T9K) (FBE = -5.3 kcal/mol).

Favourable docked pose showed interactions such as H-bonding and non-covalent interaction with amino acid residues such as leucine and cysteine.

Table S9. Computed selected structural parameters of species **I** to **V**.

Mononuclear Species																
**Cu-O4**	**Cu-N**	**Cu-O5**	**Cu-Br1**	**Cu-Br2**	**P-O1**	**P-O2**	**P-O3**	**P-C**	**∠O4-Cu-N**	**∠O4-Cu-O5**	**∠O4-Cu-X1**	**∠O4-Cu-X2**	**∠O5-Cu-X1**	**∠O5-Cu-X2**	**∠N-Cu-X1**	**∠N-Cu-X2**
Species I																
1.917	2.153	1.917	2.466	--	1.587	1.746	1.725	1.900	83.6	167.0	86.9	--	102.0	--	160.2	--
Species II																
1.929	2.305	--	2.482	2.466	1.593	1.742	1.748	1.929	89.1	--	137.2	100.0	--	--	89.3	122.0
Species III																
1.923	2.305	--	2.362	2.342	1.593	1.739	1.750	1.930	89.2	--	135.1	100.0	--	--	87.8	120.7
Dinuclear Species																
**Cu1-O1**	**Cu1-Cl1**	**Cu1-Cl2**	**Cu1-O5**	**Cu2-Cl3**	**Cu2-Cl4**	**Cu2-O4**	**C-N**	**P-C**	**∠Cl1-Cu1-Cl2**	**∠Cl1-Cu1-O5**	**∠Cl2-Cu1-O5**	**∠O1 Cu1-O5**	**∠Cl3-Cu2-Cl4**	**∠Cl3-Cu2-O4**	**∠O4-Cu2-N**	**∠Cl3-Cu2-N**
Species ^{3}IV																
1.945	2.363	2.361	1.959	2.312	2.300	2.074	1.482	1.917	173.2	92.7	93.6	174.2	104.9	148.9	88.1	89.8
Species ^{1}IV																
2.02923	2.288	2.299	2.041	2.319	2.288	2.073	1.479	1.925	107.6	143.2	90.2	91.3	105.4	144.9	88.1	89.3
Species ^{3}V																
1.945	2.365	2.362	1.945	2.451	2.417	2.117	1.479	1.916	173.4	86.4	87.0	175.5	103.8	141.4	88.3	91.9
Species ^{1}V																
1.945	2.365	2.362	1.958	2.451	2.417	2.117	1.479	1.917	173.4	86.4	87.0	175.6	103.9	141.2	88.3	91.9
